# Genome-Wide Characterization of the Aquaporin Gene Family in Radish and Functional Analysis of *RsPIP2-6* Involved in Salt Stress

**DOI:** 10.3389/fpls.2022.860742

**Published:** 2022-07-13

**Authors:** Xiaofang Yi, Xiaochuan Sun, Rong Tian, Kexin Li, Meng Ni, Jiali Ying, Liang Xu, Liwang Liu, Yan Wang

**Affiliations:** ^1^National Key Laboratory of Crop Genetics and Germplasm Enhancement, Key Laboratory of Horticultural Crop Biology and Genetic Improvement (East China), Ministry of Agriculture and Rural Affairs, College of Horticulture, Nanjing Agricultural University, Nanjing, China; ^2^College of Life Science and Food Engineering, Huaiyin Institute of Technology, Huai’an, China

**Keywords:** radish, aquaporin, PIPs, *RsPIP2-6*, salt stress

## Abstract

Aquaporins (AQPs) constitute a highly diverse family of channel proteins that transport water and neutral solutes. *AQPs* play crucial roles in plant development and stress responses. However, the characterization and biological functions of *RsAQPs* in radish (*Raphanus sativus* L.) remain elusive. In this study, 61 non-redundant members of AQP-encoding genes were identified from the radish genome database and located on nine chromosomes. Radish AQPs (RsAQPs) were divided into four subfamilies, including 21 plasma membrane intrinsic proteins (PIPs), 19 tonoplast intrinsic proteins (TIPs), 16 NOD-like intrinsic proteins (NIPs), and 5 small basic intrinsic proteins (SIPs), through phylogenetic analysis. All RsAQPs contained highly conserved motifs (motifs 1 and 4) and transmembrane regions, indicating the potential transmembrane transport function of RsAQPs. Tissue- and stage-specific expression patterns of AQP gene analysis based on RNA-seq data revealed that the expression levels of *PIPs* were generally higher than *TIPs*, *NIPs*, and *SIPs* in radish. In addition, quantitative real-time polymerase chain reaction (qRT-PCR) revealed that seven selected *RsPIPs*, according to our previous transcriptome data (e.g., *RsPIP1-3*, *1-6*, *2-1*, *2-6*, *2-10*, *2-13*, and *2-14*), exhibited significant upregulation in roots of salt-tolerant radish genotype. In particular, the transcriptional levels of *RsPIP2-6* dramatically increased after 6 h of 150 mM NaCl treatment during the taproot thickening stage. Additionally, overexpression of *RsPIP2-6* could enhance salt tolerance by *Agrobacterium rhizogenes*-mediated transgenic radish hairy roots, which exhibited the mitigatory effects of plant growth reduction, leaf relative water content (RWC) reduction and alleviation of O^2–^ in cells, as shown by nitro blue tetrazolium (NBT) staining, under salt stress. These findings are helpful for deeply dissecting the biological function of *RsAQPs* on the salt stress response, facilitating practical application and genetic improvement of abiotic stress resistance in radish.

## Introduction

Soil salinization is one of the main abiotic stressors in global agriculture production. Approximately 25% of the global cultivated land area is salinized, and the problem has sequentially deteriorated due to climatic variation and desertification (Tuteja, 2007; Zhu, 2016). Plant growth and development, as well as crop yield, are severely hindered by salt stress. An excessive soil salt content causes vegetable crops to be short, with yellow leaves and brown roots (Chrysargyris et al., 2019; Daničić et al., 2021). In addition, an unsuitable salt environment destroys the plasma membrane structure, greatly increasing membrane permeability and resulting in the destruction of the water balance in plants (Ueda et al., 2016). Osmotic stress and radial water transportation are mainly dependent on aquaporin (AQP) activity (Horie et al., 2011; Chaumont and Tyerman, 2014; Laur and Hacke, 2014; Bouda et al., 2018). AQPs are integral membrane proteins that belong to the ancient superfamily of major intrinsic proteins (MIPs), which are widely distributed in animals, plants, and microbes (Gomes et al., 2009). Increasing evidence has demonstrated that AQPs efficiently transport water and other small molecule substrates and play important regulatory roles in seed germination, tissue expansion, reproductive growth, fruit ripening, water movement, and maintenance of cellular water homeostasis in plants (Eisenbarth and Weig, 2005; Chen et al., 2013; Moshelion et al., 2015; Shivaraj et al., 2017; Zargar et al., 2017). In addition, when plants are exposed to abiotic stress, AQPs quickly respond and regulate water transport, reducing H_2_O_2_ accumulation and membrane damage by enhancing the antioxidant system in plants (Hu et al., 2012).

The typical AQPs are composed of four monomers, and each monomer contains six transmembrane domains (TM1–TM6) and five connecting loops (LA–LE), forming independent transmembrane pores localized on the intra-(LB, LD) or extracytosolic (LA, LC, LE) sides of the membrane (Afzal et al., 2016; Ozu et al., 2018). Through folding and linking, two Asn-Pro-Ala (NPA) motifs form a narrow channel to control the permeability of water (Murata et al., 2000), which plays a vital role in water molecules across the membrane. Based on protein sequence similarity and subcellular localization, AQPs are divided into eight subfamilies, including plasma membrane intrinsic proteins (PIPs), tonoplast intrinsic proteins (TIPs), NOD26-like intrinsic proteins (NIPs), small basic intrinsic proteins (SIPs), uncategorized X intrinsic proteins (XIPs), GlpF-like intrinsic proteins (GIPs), hybrid intrinsic proteins (HIPs), and large intrinsic proteins (LIPs) (Danielson and Johanson, 2008; Hussain et al., 2020). Among them, PIPs are the subfamily with the most members that can be categorized into two phylogenetic subgroups, PIP1s and PIP2s, according to the length of the N- and C-termini of PIPs (Tyerman et al., 1999). PIP2s exhibit strong water permeability when expressed in *Xenopus* oocytes, whereas PIP1s generally have much lower or even no water channel activity (Fetter et al., 2004). PIP1 and PIP2 aquaporins may interact to increase water permeability (Hachez et al., 2013). *PIP* expression levels are complexly regulated by various physiological and environmental stressors, including plant hormones and abiotic stress (Kapilan et al., 2018), especially under drought and salt stress (Srivastava et al., 2016). Overexpression of *PIP* genes can improve salt tolerance of transgenic plants in several plants, such as sugarcane (Tang et al., 2021), barley (Alavilli et al., 2016), soybean (Zhou et al., 2014), *Leymus chinensis* (Ma and Liu, 2012), durum wheat (Ayadi et al., 2011), and rice (Guo et al., 2006). *PIP* genes might function as regulators of plant salt tolerance.

Radish (*Raphanus sativus* L.) is an important root vegetable crop belonging to the *Brassicaceae* family. Soil salinization and secondary salinization causing salt stress seriously affect the yield and quality of radish taproots. However, little information on the *AQP* gene family is available on radish. In the present study, a genome-wide analysis of the identification of *AQP* genes was performed, and its evolutionary relationships, structural characteristics, promoter analysis, and chromosomal distribution were systematically characterized. Moreover, the transcript profiles of *RsPIPs* in different developmental stages and tissues are detected and seven selected genes are also performed for differentially responsive genes under salt stress. Furthermore, the biological function of *RsPIP2-6* was validated by *Agrobacterium rhizogenes*-mediated transgenic radish hairy roots in the face of salt stress. These results provide fundamental insights for the genetic improvement of salt tolerance traits and for revealing the salt stress response mechanism of radish.

## Materials and Methods

### Genome-Wide Identification of Aquaporin Genes in Radish

The gene and protein sequence information for radish were obtained from the public genome database (RGD^1^). The candidate AQP proteins that included the Asn-Pro-Ala (NPA) domain (PF00230) were identified through Pfam.^2^ The hidden Markov model (HMM) search was then processed using HMMER 3.0^3^ to retrieve the sequences, and SMART^4^ and CDD^5^ were employed to remove proteins with incomplete AQP conserved domains, ensuring the reliability of all radish aquaporin members (RsAQPs). Following this, Clustal W^6^ was conducted for multiple sequence alignment, and all AQP protein sequences, including radish and *Arabidopsis*, were imported to generate the phylogenetic tree using MEGA 5.0 with neighbor-joining (NJ) and the bootstrap value set to 1000. The *Arabidopsis* AQP protein sequences were downloaded from the TAIR database.^7^

### Chromosome Localization, Protein Properties, Gene Structure, and Promoter *Cis*-Elements Analysis

The structural intron and exon characteristics of the *RsAQP* family genes were determined using Gene Structure Display Server 2.0.^8^ The chromosome localization of *RsAQPs* was plotted using MapChart software.^9^ The ExPASy ProtParam tool^10^ was used to analyze the RsAQP protein properties, including the number of amino acids (AAs), molecular weight (MW), theoretical isoelectric point (pI), hydrophilicity index (HI) and instability index (II). The conserved motifs of the RsAQP family were identified using the MEME Suite 5.4.1.^11^ Moreover, transmembrane prediction was detected using Hidden Markov Models Server v.2.0.^12^ Additionally, the promoter region (1500 bp sequence upstream of the translation initiation sites) of *RsAQP* genes was extracted and analyzed in the PlantCARE database for the identification of potential *cis*-acting elements (Lescot et al., 2002).

### Expression Analysis of *RsAQP* Genes

The published RNA-seq data of five tissues (cortical, cambium, xylem, root tip, and leaf) at six stages (7, 14, 20, 40, 60, and 90 days after sowing) were used to analyze the expression patterns during radish development (Mitsui et al., 2015). Based on the reads per kilobase per kilo (RPKM) values, the heatmap was generated by TBtools^13^ (Chen et al., 2020). The expression profiles of the identified *RsAQP* genes under salt stress were extracted and performed from our previous transcriptome data (Sun et al., 2016).

### Plant Materials, Growth Conditions, and Salt Treatments

Two previously screened advanced inbred radish lines, namely the salt-sensitive (‘NAU-TR12’) and the salt-tolerant (‘NAU-TR17’) genotypes, were used in this study (Zhang et al., 2021). The seeds were rinsed and sterilized before germinating on moist filter paper in the dark for 2 days. Subsequently, seedlings were transferred into plastic pots and cultured at 25°C day/18°C night with 16 h light/8 h dark, 60% relative humidity and 12,000 lx light. After 3 (young seedling stage) and 8 (taproot stage) weeks, these seedlings were transferred into the plastic container with a half-strength Hoagland nutrient solution (Xu et al., 2013). During a 1-week slow seeding period, the plants were treated with 150 mM NaCl solution and the NaCl-free nutrient solution was used as a control (CK). Three biological replicates were employed in each treatment, and each replicate included 20 seedlings. Different tissues (such as leaf and root) were harvested in triplicate at 0, 6, 12, and 24 h after a continuous time under NaCl treatment. Then, the samples were immediately frozen in liquid nitrogen and subsequently stored at –80°C for further use.

### RNA Extraction and RT-qPCR Analysis

Total RNA extraction was performed with an RNAprep Pure Plant Kit (Tiangen, Beijing, China), and cDNA was synthesized using a PrimeScriptTM RT reagent kit (Takara, Dalian, China) according to the manufacturer’s instructions. RT-qPCR analysis was carried out on the LightCycler^®^ 480 System (Roche, Mannheim, Germany). All primers used for RT-qPCR are listed in Supplementary Table 3. *RsActin* was employed as the internal standard to normalize expression. The relative expression level was normalized to the *RsActin* gene and calculated using the 2^–ΔΔ*Ct*^ method (Livak and Schmittgen, 2001). Three replicates were performed in this study.

The relative expression levels of the salt stress samples were compared to those of the controls. The gene fragments for RT-qPCR were isolated among young and taproot thickening periods from two radish varieties: ‘NAU-TR12’ (salt-sensitive) and ‘NAU-TR17’ (salt-tolerant).

### *Agrobacterium rhizogenes*-Mediated Transformation System of Radish

The coding sequence (CDS) of *RsPIP2-6* was amplified with the primer pair *RsPIP2-6OE*-F*/RsPIP2-6OE*-R. The PCR fragments were then inserted between *Xba*I and *Kpn*I restriction sites (Supplementary Table 1). The plant expression vector pCambia1300 with the 35S promoter included a green fluorescent protein (GFP) tag. The recombination vector containing *RsPIP2-6* was transformed into *A*. *rhizogenes* strain MSU440.

*RsPIP2-6*-transformed radish hairy root composite plants were obtained by infection, according to Wei et al. (2016). The germinating radish seeds were sown on vermiculite and cultured at 25°C day/18°C night with 16 h light/8 h dark, 60% relative humidity and 12,000 lx light. After 4 days, seedlings with consistent growth were selected, and the original roots of the radishes were cut off. The growing tip and 0.5–1 cm elongated hypocotyl (composite plants that contained the transformed hairy roots with a wild-type shoot) were retained for *A*. *rhizogenes* infection. *Agrobacterium rhizogene* harboring *RsPIP2-6*-GFP (*OE*) or the empty vector (pCambia1300-GFP: *EV*) in 50 mL LB liquid medium plus 50 mg/L streptomycin and 100 mg/L kanamycin were incubated overnight at 28°C on a rotary shaker at 200 rpm until the OD_600_ reached 0.8–1.0 (Qin et al., 2021). Bacterial cells were centrifuged at 5000 rpm for 5 min and re-suspended in MS liquid medium (OD_600_ = 0.8–1.0) containing 100 μM acetosyringone (AS) and infected in the dark for 40–60 min (Huang et al., 2022). Subsequently, the composite plants were planted into a substrate (peat:vermiculite = 2:1) and treated with 150 mM NaCl at four leaves and one shoot period for 6 days. Three biological replicates were employed in each treatment. Each sample of at least six seedlings was harvested for salt treatment in the experiment, and three seedlings were randomly selected and photographed.

### Chlorophyll Fluorescence Measuring and Histochemical Staining

Chlorophyll fluorescence was analyzed using a chlorophyll fluorometer (IMAG-PAM). Three leaves and one shoot of soil-grown *OE* and *EV* seedlings were treated with 0 or 150 mM NaCl for 6 h before being subjected to chlorophyll fluorescence determination. The seedlings were dark-adapted for at least 30 min before measurements. Fv/Fm was averaged from equal circles of interesting areas on the leaves (Zhou et al., 2022). Chlorophyll fluorescence images and chlorophyll fluorescence parameters of the samples were measured synchronously using Imaging PAM software. Each sample of at least 9 seedlings was used for chlorophyll fluorescence determination, and one leaf was randomly selected photo. In addition, histochemical staining was conducted with NBT, as previously described by Alvarez et al. (1998), and RWC in leaves was determined according to Hu et al. (2016). Three replicates were employed in each treatment, and each replicate included at least three seedlings.

### Statistical Analysis

All experiments in this study were performed with at least three repetitions. The significance of differences determined by one-way ANOVA followed by Duncan’s test among treatment means using IBM SPSS Statistics 25 (IBM Corp., United States) was defined as significant when *P* < 0.05, as indicated in the figure legends.

## Results

### Identification and Characterization of *RsAQPs* in Radish

The homology search resulted in 62 putative AQP protein sequences obtained in radish. After removing the sequence with an incomplete NPA domain, 61 non-redundant and complete aquaporin members were identified from the radish genome database (Table 1). All members were correspondingly named according to the classification of model plant *Arabidopsis* from the TAIR database.^14^ Based on physical and chemical property analyses, the protein sizes of RsAQPs varied from 122 to 553 AAs, and 55 members (90.16% of all RsAQPs) were concentrated at 20–35 kDa. The theoretical pI values ranged from 4.96 to 10.07, and the MWs ranged from 12.76 to 61.49 kDa. Additionally, the average instability coefficient (IC) was 29.58, and most (58 members, 95.08%) were structurally stable, with an IC less than 40.00. Furthermore, all proteins except RsNIP6-3 were predicted to be hydrophobic.

**TABLE 1:** Identification and characterization of AQP proteins in radish.

Protein name	Gene ID	Number of amino acids	Molecular weight	Theoretical pI	Instability index	Aliphatic index	Hydropathy index
*RsPIP1-1*	Rs265710	286	30667.57	8.86	31.02	96.92	0.365
*RsPIP1-2*	Rs218100	286	30614.59	9.01	34.55	96.92	0.378
*RsPIP1-3*	Rs605220	286	30527.6	9.16	31.22	97.62	0.419
*RsPIP1-4*	Rs212290	286	30527.6	9.16	31.22	97.62	0.419
*RsPIP1-5*	Rs000570	286	30588.65	9.02	32.08	94.55	0.386
*RsPIP1-6*	Rs480800	286	30620.65	9.03	32.63	94.5	0.376
*RsPIP1-7*	Rs159240	287	30749.77	8.99	29.48	92.16	0.359
*RsPIP2-1*	Rs359040	283	21453.85	6.71	32.14	95.54	0.445
*RsPIP2-2*	Rs359080	283	30119.75	6.51	30.33	95.51	0.501
*RsPIP2-3*	Rs359050	285	30232.87	6.95	30.86	96.21	0.505
*RsPIP2-4*	Rs612380	285	30232.87	6.95	30.86	96.21	0.505
*RsPIP2-5*	Rs120730	283	30039.7	6.51	29.65	97.6	0.525
*RsPIP2-6*	Rs257780	287	30461.24	6.5	34.15	99.62	0.563
*RsPIP2-7*	Rs404730	285	30099.91	7.62	26.34	103.75	0.522
*RsPIP2-8*	Rs079440	283	30067.94	8.53	28.56	100.04	0.475
*RsPIP2-9*	Rs137470	285	30061.83	6.88	28.35	102.95	0.505
*RsPIP2-10*	Rs123510	288	30907.89	8.97	25.25	102.95	0.477
*RsPIP2-11*	Rs260210	202	21453.85	6.71	32.14	95.54	0.445
*RsPIP2-12*	Rs151510	281	29853.65	8.82	26.49	96.9	0.427
*RsPIP2-13*	Rs044090	282	29837.71	8.83	29	97.62	0.493
*RsPIP2-14*	Rs430170	281	29810.69	8.99	31.62	96.23	0.471
*RsTIP1-1*	Rs204560	251	25610.7	6.02	26.19	107.73	0.797
*RsTIP1-2*	Rs176140	253	25832.86	5.61	25.55	110.71	0.816
*RsTIP1-3*	Rs316110	253	25734.72	5.32	30.58	111.9	0.834
*RsTIP1-4*	Rs316050	253	25734.72	5.32	30.58	111.9	0.834
*RsTIP1-5*	Rs105440	252	25903.02	5.12	16.56	106.51	0.817
*RsTIP1-6*	Rs480080	252	25943.02	5.13	20.46	104.96	0.808
*RsTIP2-1*	Rs232070	248	24886.86	5.32	26.03	110.6	0.956
*RsTIP2-2*	Rs301510	249	25020.13	5.32	29.77	114.1	1.001
*RsTIP2-3*	Rs301530	249	25020.13	5.32	29.77	114.1	1.001
*RsTIP2-4*	Rs282040	248	24852.92	5.3	23.21	113.39	0.993
*RsTIP2-5*	Rs037700	217	22021.56	6.03	20.97	110.65	0.811
*RsTIP2-6*	Rs180310	138	14082.46	5.12	29.4	114.49	0.808
*RsTIP2-7*	Rs321260	145	14486.85	4.96	23.69	125.79	1.084
*RsTIP2-8*	Rs060660	465	46575.37	5.05	22.76	119.18	1.082
*RsTIP3-1*	Rs455830	267	28168.67	7.2	25.9	111.16	0.606
*RsTIP3-2*	Rs299110	267	28468.07	6.54	31.22	112.66	0.581
*RsTIP3-3*	Rs013400	268	28676.32	6.49	28.67	112.54	0.568
*RsTIP4-1*	Rs194740	249	26195.44	5.3	23	112.81	0.726
*RsTIP5-1*	Rs345340	255	26402.72	6.71	25.98	96.35	0.759
*RsNIP1-1*	Rs597390	297	31511.65	8.62	31.71	107.68	0.446
*RsNIP1-2*	Rs051540	297	31511.65	8.62	31.71	107.68	0.446
*RsNIP1-3*	Rs162110	289	30633.6	8.86	29.28	105.92	0.469
*RsNIP2-1*	Rs255960	282	30253.84	8.66	40.63	111.12	0.242
*RsNIP2-2*	Rs444150	324	34586.75	5.75	34.68	94.78	0.318
*RsNIP2-3*	Rs249950	323	34727.96	6.42	41.97	101.73	0.326
*RsNIP4-1*	Rs186920	283	30281.59	7.66	33.43	105.05	0.575
*RsNIP4-2*	Rs510390	278	29678.07	8.6	31.55	111.12	0.745
*RsNIP4-3*	Rs580980	283	30086.34	8.21	30.21	110.88	0.689
*RsNIP4-4*	Rs552680	283	30120.35	6.81	31.12	112.26	0.707
*RsNIP5-1*	Rs090820	301	31073.22	8.66	35.28	96.31	0.537
*RsNIP6-1*	Rs103230	305	31823.04	8.26	33.09	99.87	0.429
*RsNIP6-2*	Rs103190	242	24968.15	7	27.15	102.85	0.594
*RsNIP6-3*	Rs103210	553	61490.11	5.85	31.64	90.98	−0.265
*RsNIP7-1*	Rs222440	127	13465.47	5.68	39.16	108.9	0.54
*RsNIP7-2*	Rs222590	122	12759.07	8.8	44.72	122.21	0.829
*RsSIP1-1*	Rs221110	239	25576.16	9.68	27.73	101.8	0.687
*RsSIP1-2*	Rs291150	255	27481.6	10.07	26.94	96.9	0.459
*RsSIP2-1*	Rs536450	237	25738.68	9.75	29.26	122.49	0.75
*RsSIP2-2*	Rs374490	238	26159.29	9.7	24.86	117.06	0.656
*RsSIP2-3*	Rs515300	237	25815.76	9.61	20.08	115.11	0.664

### Phylogenetic Analysis of *RsAQP* Genes

**FIGURE 1:**
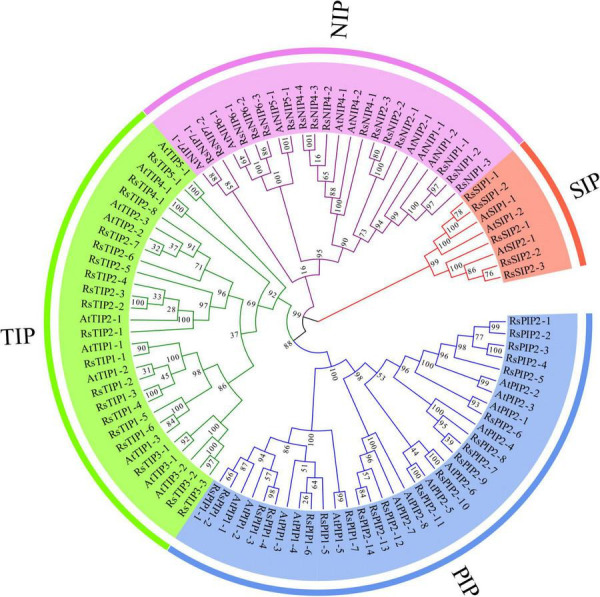
Phylogenetic relationship between the members of RsAQP and AtAQP.

To systematically classify the subfamily of RsAQPs and reveal the evolutionary relationship with the aquaporin members of *Arabidopsis* (AtAQP), a phylogenetic tree was constructed using the neighbor-joining method with the amino acid sequences (Figure 1). By homologue comparative analysis of the protein sequences between RsAQPs and AtAQPs, the 61 RsAQPs were separated into four distinct subfamilies according to their grouping with AtAQPs, covering RsPIPs, RsTIPs, RsNIPs, and RsSIPs. Among them, RsPIPs were the most abundant subfamily, containing 21 members, which were further divided into 2 subgroups containing 7 RsPIP1 members and 14 RsPIP2 members. There were 19 members involved in RsTIPs and 5 members in RsSIPs, which were clustered into 5 and 2 subgroups, respectively. The orthologous sequence of AtNIP3-1 was not identified in radish.

### Gene Structure and Conserved Domain Analysis of RsAQPs

**FIGURE 2:**
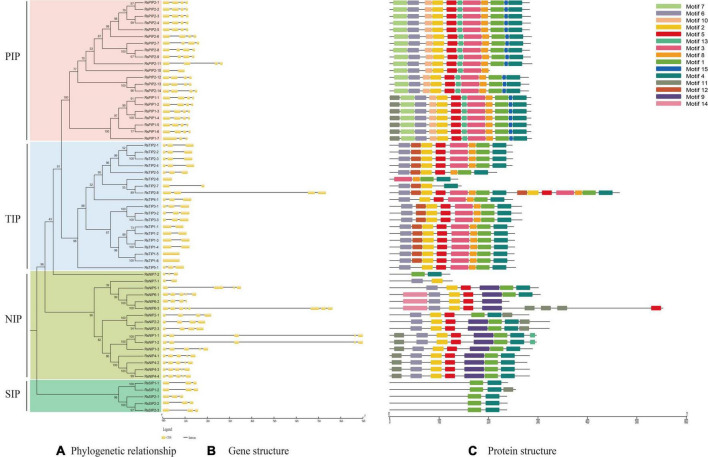
Conserved motifs and gene structure distribution of RsAQP proteins. **(A)** Phylogenetic tree of RsAQP proteins. **(B)** Exon–intron structure of *AQP* genes in radish. **(C)** Conserved motif distribution of RsAQP proteins.

Exon–intron organization analysis of the 61 RsAQPs showed that the number of introns ranged from zero to seven, and the same subfamily generally contained similar gene structures (Figures 2A,B). Specifically, the RsSIP subfamily contained two introns, while the RsPIP subfamily displayed three introns, except for *RsPIP1-7* and *RsPIP2-10*, which had two and one introns, respectively. Most of the *RsTIPs* had two introns, except *RsTIP1-5* and *RsTIP1-6*, which lacked introns. The structure of the RsNIP subfamily was relatively complex, with the number of introns varying from one to seven.

A total of 15 conserved motifs were generated from 61 RsAQPs (Figure 2C), and the motif compositions were similar in the same subfamily. Among these, motifs 1 and 4 were involved in all RsAQP proteins, suggesting that these motifs were the basic region of RsAQPs. However, some motifs were unique and were only detected in specific subfamilies. For instance, motifs 7, 10, and 15 were detected only in RsPIPs, whereas motifs 9 and 12 were uniquely distributed in RsNIPs and RsTIPs, respectively. These special motifs might be the characteristic domains of RsPIPs, RsTIPs, and RsNIPs. In addition, some motifs were covered in different subfamilies. For example, motifs 2, 5, and 6 could be discovered in RsPIPs, RsTIPs, and RsNIPs, while motifs 3 and 8 were both distributed in RsPIPs and RsTIPs. The diversity of motif compositions in the RsAQPs family reflected their evolutionary processes and contributed to their functional differentiation.

### Promoter *Cis*-Element Prediction and Transmembrane Region Analysis

**FIGURE 3:**
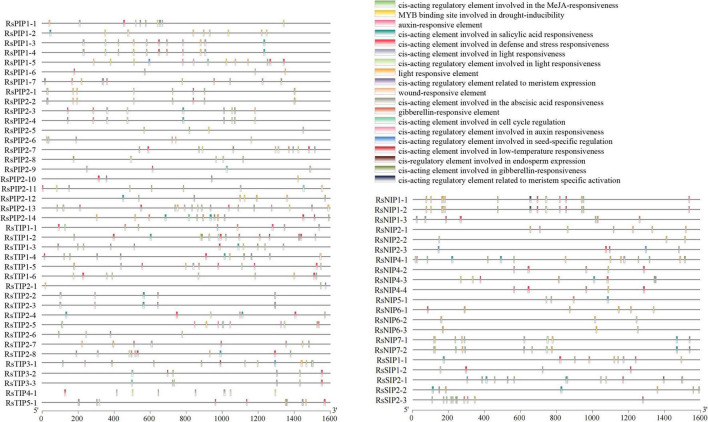
Promoter *cis*-element prediction of *RsAQP* genes.

Various *cis*-acting elements, including stress-, development-, and hormone-responsive elements, were widely distributed in the promoter regions of the *RsAQP* genes (Figure 3). By calculating the number of different *cis*-elements, the light-responsive element was the most frequent in the *RsAQP* promoter, followed by MeJA-responsive and abscisic acid-responsive elements. Notably, defense and stress elements were distributed in all RsAQP subfamilies. The wound-responsive element only existed in the *RsPIP* and *RsTIP* promoters, while the element involved in seed-specific regulation was only present in the *RsSIPs*. Moreover, none of the elements involved in cell cycle regulation were contained in the *RsNIPs* and *RsSIPs* (Table 2). These results suggest that the transcriptional regulation of different types of *RsAQP* genes was diverse, indicating the diversity of RsAQP functions. Furthermore, other *cis*-elements involved in osmotic stress, such as MBS (CAACTG), ABRE (ACGTG) and ABA (TAACCA), were also observed in R*sAQP* promoters. This suggests that these aquaporin members may be regulated by various factors in radish, including drought and ABA, which need to be experimentally demonstrated in further studies. Moreover, all RsAQPs contained transmembrane regions that varied from 3 to 12 (Supplementary Table 1), and more than half (33 RsAQPs) comprised six typical transmembrane domains.

**TABLE 2:** Number of occurrences of each *cis*-acting element in the *RsAQP* promoter.

Responsive elements	*Cis*-element	Occurrences	Total
Hormone	MeJA-responsive element	162	352
	Auxin-responsive element	28	
	Salicylic acid-responsive element	28	
	Abscisic acid-responsive element	102	
	Gibberellin-responsive element	32	
Stress	Drought-inducibility	30	105
	Defense and stress-responsive element	36	
	Wound-responsive element	3	
	Low-temperature-responsive element	36	
Development	Light-responsive element	230	266
	Meristem expression element	22	
	Cell cycle regulation element	7	
	Seed-specific regulation element	4	
	Endosperm expression element	3	

### Chromosomal Localization Analysis of RsAQPs

**FIGURE 4:**
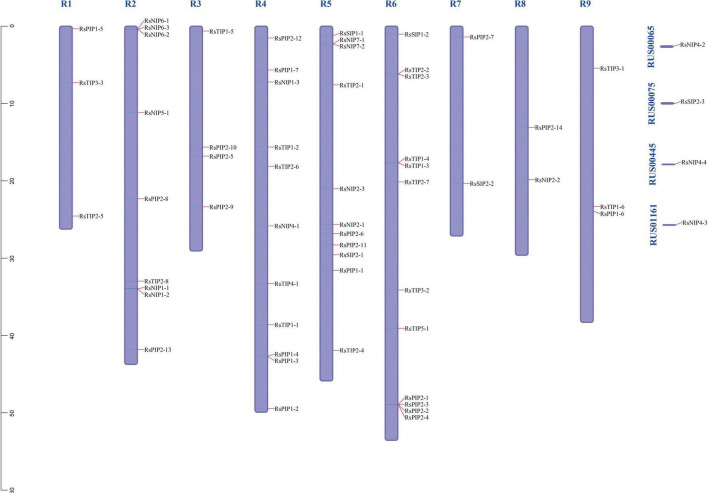
Chromosomal distributions of *RsAQP* genes.

A total of 57 *RsAQPs* (93.44%) were successfully located on nine chromosomes of radish through MapChart analysis, except for *RsSIP2-3*, *RsNIP4-2*, *RsNIP4-3*, and *RsNIP4-4* (Figure 4 and Supplementary Table 2). At least two members were mapped on each chromosome. Interestingly, some *RsAQP*s were located in clusters in certain chromosomal regions, especially on chromosomes 2 and 6. Among them, chromosome 6 possessed the largest number of *RsAQP* genes, followed by chromosomes 4 and 5, and the fewest number of *RsAQP* genes were found on chromosomes 7 and 8.

### Spatial and Temporal Expression Patterns of RsAQPs

**FIGURE 5:**
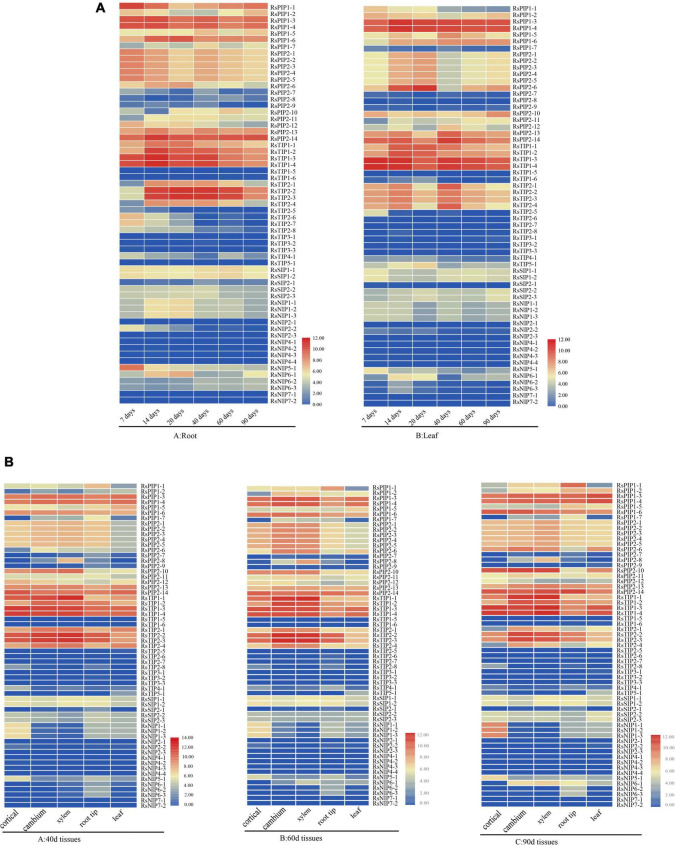
Expression profiles of *RsAQP* genes in different stages and tissues. **(A)** *RsAQP* gene expression heatmap in six stages (7, 14, 20, 40, 60, and 90 days) of two tissues (root and leaf). **(B)** *RsAQP* gene expression heatmap in three stages (40, 60, and 90 days) of five tissues (cortical, cambium, xylem, root tip, and leaf).

The expression profiles of the 61 *RsAQP* genes among different tissues (cortical, cambium, xylem, root tip, and leaf) and developmental stages (40, 60, and 90 days) were determined in the publicly available RNA-seq data (Mitsui et al., 2015) and presented in the heatmap (Figure 5). In total, the expression levels of *RsPIPs* and *RsTIPs* were significantly higher than those of *RsNIPs* and *RsSIPs* in all tissues. For the *RsTIP* subfamily, *RsTIP1-1* to *RsTIP1-4*, *RsTIP2-2*, and *RsTIP2-3* showed high expression within roots and leaves, while other *RsTIP* members were expressed at extremely low levels. However, most *RsPIPs* showed high transcript levels in the leaves and roots of the radish, especially *RsPIP2s*. For example, *RsPIP2-1*, *RsPIP2-2*, *RsPIP2-3*, *RsPIP2-4* and *RsPIP2-5* maintained relatively high expression levels at the middle stage of the roots, while the expression patterns of *RsPIP2-6* were relatively higher at the earlier and later stages (Figure 5A).

In the tissues for 40, 60, and 90 days, the expression levels of *RsPIPs* and *RsTIPs* were also significantly increased compared to *RsNIPs* and *RsSIPs*. For the *RsTIP* subfamily, *RsTIP1-1* to *RsTIP1-4* and *RsTIP2-1* to *RsTIP2-4* were expressed at high levels. In the *RsPIP* subfamily, *RsPIP1-3*, *RsPIP1-4*, *RsPIP1-6*, *RsPIP2-13*, and *RsPIP2-14* were highly expressed in the cortex, cambium, xylem, root tip, and leaf. *RsPIP2-6* was mainly expressed in the cortex, cambium and xylem, while *RsPIP2-1* was intensively expressed in the cambium and xylem (Figure 5B). These *RsPIP* genes might play critical roles in the development of radish roots.

### Expression Profiles of *RsPIPs* in Different Stages and Varieties Under Salt Stress

**FIGURE 6:**
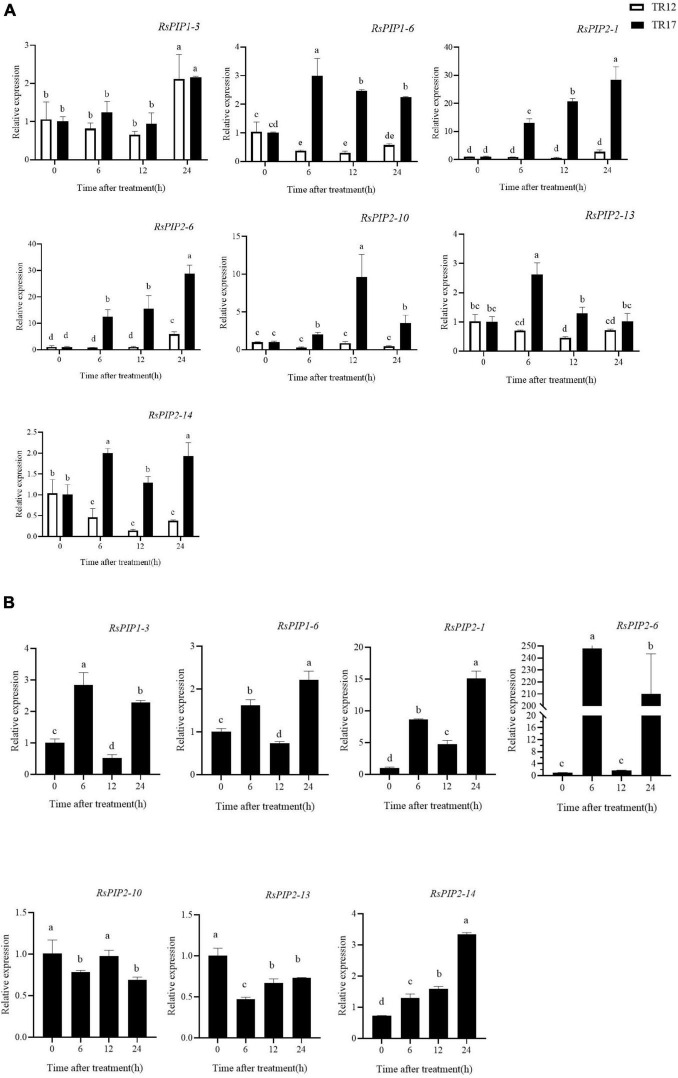
Expression levels of *RsPIP* genes under NaCl treatment in young roots and the taproot thickening period. **(A)** Expression levels of *RsPIP* genes in young roots for the indicated time (h) under 150 mM NaCl treatment. **(B)** Expression levels of *RsPIP* genes in the taproot thickening period for the indicated time (h) under 150 mM NaCl treatment. *RsActin* was used as an internal control for qRT-PCR. The relative expression levels of the *RsPIP* genes were calculated based on the comparative threshold cycle (Ct). Statistical analysis was processed using GraphPad Prism 8. The significant difference was analyzed using IBM SPSS Statistics 25, values with different lowercase letters indicate a significant difference at *p* < 0.05 according to Duncan’s multiple range tests. Each bar shows the mean ± SE of the triplicate assay.

Based on our previous RNA-seq data in radish taproots and the variation of the expression levels under salt stress (Xie et al., 2015; Sun et al., 2016), seven *RsPIPs* (*RsPIP1-3*, *1-6*, *2-1*, *2-6*, *2-10*, *2-13*, and *2-14*) were selected to further determine their expression patterns by RT-qPCR under different salt exposure durations in two radish varieties (Figure 6 and Supplementary Tables 3, 4). At the seeding stage, almost all seven *RsPIP* genes were significantly upregulated under salt stress in the salt-tolerant variety ‘NAU-TR17,’ however, they did not show obvious variation in the salt-sensitive variety ‘NAU-TR12’ (Figure 6A). The salt-responsive expression profiles of these genes were screened at the taproot thickening period in ‘NAU-TR17.’ As shown in Figure 6B, the *RsPIP2-1* and *RsPIP2-6* genes exhibited sharp growth at 6 and 24 h, especially for *RsPIP2-6*, with a 250-fold increase.

### *Agrobacterium rhizogenes*-Mediated Overexpression of *RsPIP2-6* Confers Salt Tolerance in Radish With Transgenic Hairy Roots

**FIGURE 7:**
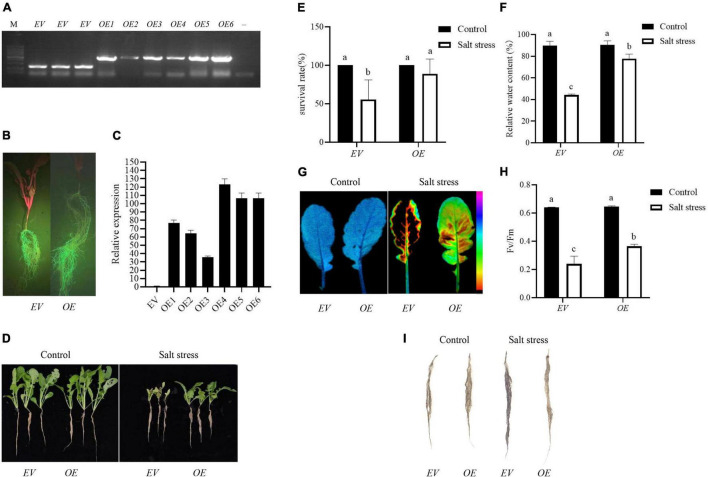
Overexpression of *RsPIP2-6* positively regulated salt tolerance in radish. **(A)** PCR identification of *RsPIP2-6*-GFP (1300-GFP-F/*RsPIP2-6*-GFP-R primers) and 1300-GFP (1300-GFP-F/1300-GFP-R primers) in transgenic radish hairy roots. M: DL5000 marker; –, ddH_2_O; *EV*: radish hairy root containing empty vector (pCambia1300-GFP), *OE*: radish hairy root overexpressing *RsPIP2-6*. **(B)** Green fluorescent protein (GFP) fluorescence in the hairy roots of radish. **(C)** Relative expression levels of *RsPIP2-6* in *EV* and *OEs*. **(D)** Phenotypes of *EV* and *OE* seedlings with 0 or 150 mM NaCl (150 mM for 6 days). **(E)** Statistical analysis of the survival rates of *EV*s and *OE*s with 0 or 150 mM NaCl. **(F)** Relative water content of *EV*s and *OE*s in radish with 0 or 150 mM NaCl. **(G)** Lipid peroxidation visualized by autoluminescence imaging. The color palette indicated luminescence intensity from low (purple) to high (black) values. **(H)** Fv/Fm rate in *EV*s and *OE*s radish with 0 or 150 mM NaCl. **(I)** Histochemical staining with NBT in the hairy roots of radish *EVs* and *OEs* with 0 or 150 mM NaCl. Each bar shows the mean ± SE of the triplicate assay, values with different lowercase letters indicate a significant difference at *p* < 0.05 according to Duncan’s multiple range tests.

*Agrobacterium rhizogenes*-mediated transformation was employed to determine the biological gene function of *RsPIP2-6* in radish when exposed to salt stress, based on the transcript expression level. *RsPIP2-6*-overexpressing hairy roots were successfully obtained, and transgenic positive hairy roots were identified by PCR, GFP signal detection and RT-qPCR (Figures 7A–C). The composite plants of *OE* with high expression in hairy roots were used for functional verification, while transgenic hairy root *EV* were used as a control. As shown in Figure 7D, no significant phenotypic differences were observed between the *EV* and *OE* plants under normal conditions. After exposure to 150 mM NaCl solution for 6 days, the leaves of *EV* plants were severely withered and yellowed or were dead and had a lower RWC in the leaves, while *OE* plants still grew vigorously and had a higher leaf RWC (Figures 7E,F). Additionally, the survival rate of *EV* plants was reduced to 55.5%, while *OE* exhibited a reduction of 88.8% compared to their untreated conditions. Interestingly, the lateral root numbers of *OE* were significantly more plentiful than *EV*. The FluorCam chlorophyll fluorescence imaging system showed that the fluorescence intensity of *EV* plants markedly decreased in comparison to transgenic plants during salt stress (Figure 7G), indicating that photosynthetic capacity (Fv/Fm) had a downward trend. The photosynthetic capacity of transgenic plants was higher than that of *EV* plants, which indicated that *OE* could alleviate the damage caused by salt stress on photosynthesis and could improve the salt tolerance of radish (Figure 7H). NBT staining showed that *EV* exhibited more severe damage in comparison with *OE* roots under salt stress (Figure 7I). Taken together, these results indicate that *RsPIP2-6* might be a positive regulator in radish against salt stress.

## Discussion

### Characterization of *AQP* Gene Family Members in Radish

The AQPs, as a class of multifunctional proteins, not only participate in maintaining cellular water homeostasis in plants but also in other physiological activities, such as seed germination, growth and development, transport of nutrient elements, heavy metal elements, CO_2_ transport, and stomatal movement, especially abiotic stress tolerance (Martinez-Ballesta and Carvajal, 2014). Accurate annotation of the *AQP* gene was an important starting point for future research on the gene function of analysis. An increasing number of *AQP* genes have been identified in many plants *via* genome sequencing. The *AQP* gene family has 39 members in *Arabidopsis* (Johanson et al., 2001), 42 in apple (Liu et al., 2019), 59 in *Brassica rapa* (Kayum et al., 2017), 33 in rice (Nguyen et al., 2013), 76 in tobacco (De Rosa et al., 2020), 47 in tomato (Reuscher et al., 2013), and 40 in chickpea (Deokar and Tar’an, 2016). However, the number and molecular characteristics of *AQP* family genes in radish are largely unclear. In the present study, 61 *AQP* genes were identified by whole genome analysis of *AQP*-encoding genes in radish. A higher number of *RsAQP* genes might indicate specific amplification, with higher evolution and more meticulous functional division. The RsAQP family was divided into four subfamilies (PIP, TIP, NIP, and SIP) based on their homology to AtAQPs. Interestingly, there were generally more members of each subfamily of radish than *Arabidopsis*, but no homologous genes of *AtNIP3-1* were identified in radish. The gene number of the *PIP* subfamily was significantly higher than that of other subfamilies in most plants, including radish, which indicated that *PIPs* had a more complex evolutionary process. Additionally, all AQPs in *B*. *rapa* functional analysis showed that most PIP subfamily proteins exhibited a high degree of identity with abiotic stress-related AQP proteins from other plant species (Kayum et al., 2017). The phylogenetic relationship of RsAQPs was also supported by both their gene structures and conserved motifs. From an evolutionary perspective, the increasing number of genes might be due to gene replication events, including segmental and tandem duplication (Bancroft, 2001). Gene structure analysis showed that each subfamily displayed a similar exon–intron organization in *Arabidopsis* and radish (Jiang et al., 2020). Nineteen *RsPIP* genes contained three introns, aside from *RsPIP1-7* and *RsPIP2-10*. *RsTIPs* possessed introns, with numbers varying from zero to three, which was also similar to *AtTIPs*. Introns are related to gene evolution, which has been proposed to affect gene expression (Rose, 2008). More and longer introns exist in more highly expressed genes (Ren et al., 2006). The gain/loss of exons and introns might be the result of chromosomal rearrangements and fusions and can potentially lead to the functional diversification of multiple gene families (Xu et al., 2012).

The expression of *AQP* genes is regulated by various stressors in plants, such as drought, salt, and cold (Feng et al., 2018; Pawłowicz and Masajada, 2019). Promoter analysis revealed that the RsAQP gene promoters contained *cis*-elements in response to multiple hormones, stress, and development (Table 2). Subsequently, the expression of seven *RsPIP* genes was upregulated under salt exposure, indicating that they might play a crucial role in the response to salt stress. Similar results were also observed in soybean (Zhou et al., 2014), *Arabidopsis* (Feng et al., 2018), and *Canavalia rosea* (Lin et al., 2021). The distribution of *RsAQP* in linkage groups showed tandem duplicated pairs, such as *RsPIP2-1*, *RsPIP2-2*, *RsPIP2-3*, and *RsPIP2-4*, on the R6 chromosome, which might have been caused by gene duplication during evolution. Tandem duplications are a common phenomenon in nature, such as leucine-rich repeat domains in asparagus with both tandem genes and duplication across multiple chromosomes (Die et al., 2018). Conserved motif analysis showed that all RsAQP proteins shared the typical AQP domain. Motifs 1 and 4 were distributed in the four subfamilies (PIP, TIP, NIP, and SIP), indicating that they were highly conserved and might be the characteristic domain of the RsAQP family. Motifs 9 and 12 were distributed only in the TIP and NIP subfamilies, respectively.

### Expression Divergence of *RsAQP* Genes

The expression level of *AtPIP2* was downregulated under salt stress in the roots of *Arabidopsis* (Boursiac et al., 2005), while *OsPIP2* was upregulated in rice (Guo et al., 2006). In the present study, *RsPIP2-6* increased dramatically compared to other *RsPIP* genes in the taproot thickening period of ‘NAU-TR17’ under salt stress. Therefore, *RsPIP2-6* might be a critical candidate gene for salt tolerance. Each specific isoform, as well as the plant genotype, might influence transcriptional aquaporin regulation under salt stress in broccoli plants (Muries et al., 2011). *FaPIP1;2* and *FaTIP1;1* transcript levels increased after salt treatment in a highly salt-tolerant genotype, whereas *FaPIP2;1* remained a relatively stable transcript level (Pawłowicz et al., 2017). The transcription level of the *PIP2;4* gene increased, while the *PIP1;2*, *TIP1;1*, and *TIP2;2* genes were reduced under salinity stress in *Piriformospora indica* (Ghorbani et al., 2019). The seedlings and reproductive stages were more vulnerable to salt stress than the vegetative stages, while the roots were more sensitive than other organs (Nam et al., 2015). These studies suggested that AQPs from different species had a high sequence homology, whereas they retained functional and regulatory specificity. These different, even contradictory, transcriptional regulations of *AQPs* might be caused by the tissue location of *AQPs*, plant species and growth phase, and salt concentration and duration of treatment.

The high efficiency of genetic transformation is an indispensable factor in gene function verification and germplasm improvement in radish. However, the efficiency of *A*. *tumefaciens*-mediated transformation in radish is extremely low, which greatly hinders gene function analysis (Muto et al., 2021). Therefore, the high-throughput production of transgenic plants in the short run is important for gene function research, especially for plants with a “bottleneck” to plant regeneration (Jian et al., 2009). To date, a fast and efficient transformation technique with *A*. *rhizogenes* has been widely used for functional genomics in plants (An et al., 2017; Che et al., 2019; Qin et al., 2021). In radish, only two reports have been successful in developing transgenic plants using the *A*. *rhizogenes*-mediated method (Tanaka et al., 1985; Balasubramanian et al., 2018). Here, *A*. *rhizogenes*-mediated transformation using composite plants as explants was performed to determine the overexpression of *RsPIP2-6* in radish. As a result, *RsPIP2-6*-transformed plants grew more vigorously, with a higher survival rate and a lower degree of damage compared with empty vector-transformed plants under salt stress. In a recent report, overexpression of *IbPSS1* improved salt tolerance in transgenic sweet potato lines obtained from an *A*. *rhizogenes*-mediated transformation system (Yu et al., 2020). *GmLecRlk*-overexpressing soybean lines have significantly enhanced salt tolerance by *A*. *rhizogenes* (Zhang et al., 2022). Similar to the above results, *RsPIP2-6* could also improve radish tolerance to salt stress using the *A*. *rhizogenes*-mediated transformation system. This finding provides a new idea for the breeding of genetically modified radish.

## Conclusion

In this study, 61 *RsAQP* genes were identified and characterized based on radish genome data. Furthermore, phylogenetic analysis, gene structure, conserved motifs, promoter *cis*-elements, chromosome distribution, and RNA-seq expression analysis of RsAQP were conducted. The expression profiles of *RsPIPs* in different stages and tissues under salt stress indicate that *PIPs* might play a vital role in maintaining the water potential homeostasis of radish exposed to salt stress. In addition, overexpression of *RsPIP2-6* could enhance salt tolerance by *Agrobacterium rhizogenes*-mediated transgenic radish hairy roots, which showed enhanced tolerance to salt stress. These results provide a beneficial resource for the evolution and function of *RsAQPs* and provide a basis for the breeding and genetic engineering of radish.

## Data Availability Statement

The original contributions presented in the study are included in the article/Supplementary Material, further inquiries can be directed to the corresponding author.

## Author Contributions

XY and YW conceived and designed the study. JY and RT contributed to data collection and bioinformatics analysis. KL, XS, and MN were responsible for sample collection and RT-qPCR analysis. XY and XS drafted the manuscript and prepared the figures. LX and LL were contributed to revising the manuscript. All authors read and approved the final manuscript.

## Conflict of Interest

The authors declare that the research was conducted in the absence of any commercial or financial relationships that could be construed as a potential conflict of interest.

## Publisher’s Note

All claims expressed in this article are solely those of the authors and do not necessarily represent those of their affiliated organizations, or those of the publisher, the editors and the reviewers. Any product that may be evaluated in this article, or claim that may be made by its manufacturer, is not guaranteed or endorsed by the publisher.

## Footnotes

1
http://radish-genome.org/

2
http://pfam.xfam.org

3
http://hmmer.janelia.org/

4
http://smart.embl-heidelberg.de/

5
https://www.ncbi.nlm.nih.gov/Structure/cdd/wrpsb.cgi

6
https://pir.georgetown.edu/pirwww/search/multialn.shtml

7
https://www.arabidopsis.org/

8
http://gsds.cbi.pku.edu.cn/

9
https://mapchart.net/greece.html

10
https://www.expasy.org/

11
https://meme-suite.org/meme/

12
https://services.healthtech.dtu.dk

13
https://github.com/CJ-Chen/TBtools

14
http://www.arabidopsis.org

## References

[B1] Afzal Z. Howton T. Sun Y. Mukhtar M. (2016). The roles of aquaporins in plant stress responses. *J. Dev. Biol.* 4:9. 10.3390/jdb4010009 29615577 PMC5831814

[B2] Alavilli H. Awasthi J. P. Rout G. R. Sahoo L. Lee B. H. Panda S. K. (2016). Overexpression of a barley aquaporin gene, HvPIP2;5 confers salt and osmotic stress tolerance in yeast and plants. *Front. Plant Sci.* 7:1566. 10.3389/fpls.2016.01566 27818670 PMC5073208

[B3] Alvarez M. E. Pennell R. I. Meijer P. J. Ishikawa A. Dixon R. A. Lamb C. (1998). Reactive oxygen intermediates mediate a systemic signal network in the establishment of plant immunity. *Cell* 92 773–784. 10.1016/s0092-8674(00)81405-1 9529253

[B4] An J. Hu Z. Che B. Chen H. Yu B. Cai W. (2017). Heterologous expression of Panax ginseng PgTIP1 confers enhanced salt tolerance of soybean cotyledon hairy roots, composite, and whole plants. *Front. Plant Sci.* 8:1232. 10.3389/fpls.2017.01232 28769947 PMC5512343

[B5] Ayadi M. Cavez D. Miled N. Chaumont F. Masmoudi K. (2011). Identification and characterization of two plasma membrane aquaporins in durum wheat (*Triticum turgidum* L. subsp. durum) and their role in abiotic stress tolerance. *Plant Physiol. Biochem.* 49 1029–1039. 10.1016/j.plaphy.2011.06.002 21723739

[B6] Balasubramanian M. Anbumegala M. Surendran R. Arun M. Shanmugam G. (2018). Elite hairy roots of Raphanus sativus (L.) as a source of antioxidants and flavonoids. *3 Biotech* 8:128. 10.1007/s13205-018-1153-y 29450118 PMC5811410

[B7] Bancroft I. (2001). Duplicate and diverge: the evolution of plant genome microstructure. *Trends Genet.* 17 89–93. 10.1016/s0168-9525(00)02179-x 11173118

[B8] Bouda M. Brodersen C. Saiers J. (2018). Whole root system water conductance responds to both axial and radial traits and network topology over natural range of trait variation. *J. Theor. Biol.* 456 49–61. 10.1016/j.jtbi.2018.07.033 30055183

[B9] Boursiac Y. Chen S. Luu D. T. Sorieul M. van den Dries N. Maurel C. (2005). Early effects of salinity on water transport in Arabidopsis roots. Molecular and cellular features of aquaporin expression. *Plant Physiol.* 139 790–805. 10.1104/pp.105.065029 16183846 PMC1255996

[B10] Chaumont F. Tyerman S. D. (2014). Aquaporins: highly regulated channels controlling plant water relations. *Plant Physiol.* 164 1600–1618. 10.1104/pp.113.233791 24449709 PMC3982727

[B11] Che B. Cheng C. Fang J. Liu Y. Jiang L. Yu B. (2019). The Recretohalophyte Tamarix TrSOS1 gene confers enhanced salt tolerance to transgenic hairy root composite cotton seedlings exhibiting virus-induced gene silencing of GhSOS1. *Int. J. Mol. Sci.* 20:2930. 10.3390/ijms20122930 31208046 PMC6628528

[B12] Chen C. Chen H. Zhang Y. Thomas H. R. Frank M. H. He Y. (2020). TBtools: an integrative toolkit developed for interactive analyses of big biological data. *Mol. Plant.* 13 1194–1202. 10.1016/j.molp.2020.06.009 32585190

[B13] Chen W. Yin X. Wang L. Tian J. Yang R. Liu D. (2013). Involvement of rose aquaporin RhPIP1;1 in ethylene-regulated petal expansion through interaction with RhPIP2;1. *Plant Mol. Biol.* 83 219–233. 10.1007/s11103-013-0084-6 23748738

[B14] Chrysargyris A. Papakyriakou E. Petropoulos S. A. Tzortzakis N. (2019). The combined and single effect of salinity and copper stress on growth and quality of *Mentha spicata* plants. *J. Hazard. Mater.* 368 584–593. 10.1016/j.jhazmat.2019.01.058 30716568

[B15] Daničić M. Maksimović I. Putnik-Delić M. Kastori R. Crnobarac J. Jaćimović G. (2021). Biomass production and mineral composition of coriander (*Coriandrum sativum* L.) exposed to NaCl. *Biol. Futur.* 72 453–459. 10.1007/s42977-021-00090-4 34554488

[B16] Danielson J. A. Johanson U. (2008). Unexpected complexity of the aquaporin gene family in the moss *Physcomitrella patens*. *BMC Plant Biol.* 8:45. 10.1186/1471-2229-8-45 18430224 PMC2386804

[B17] De Rosa A. Watson-Lazowski A. Evans J. R. Groszmann M. (2020). Genome-wide identification and characterisation of aquaporins in *Nicotiana tabacum* and their relationships with other Solanaceae species. *BMC Plant Biol.* 20:266. 10.1186/s12870-020-02412-5 32517797 PMC7285608

[B18] Deokar A. A. Tar’an B. (2016). Genome-wide analysis of the aquaporin gene family in chickpea (*Cicer arietinum* L.). *Front. Plant Sci.* 7:1802. 10.3389/fpls.2016.01802 27965700 PMC5126082

[B19] Die J. V. Castro P. Millán T. Gil J. (2018). Segmental and tandem duplications driving the recent NBS-LRR gene expansion in the asparagus genome. *Genes* 9:568. 10.3390/genes9120568 30477134 PMC6316259

[B20] Eisenbarth D. A. Weig A. R. (2005). Dynamics of aquaporins and water relations during hypocotyl elongation in *Ricinus communis* L. seedlings. *J. Exp. Bot.* 56 1831–1842. 10.1093/jxb/eri173 15897227

[B21] Feng Z. Xu S. Liu N. Zhang G. Hu Q. Xu Z. (2018). Identification of the AQP members involved in abiotic stress responses from *Arabidopsis*. *Gene.* 646 64–73. 10.1016/j.gene.2017.12.048 29278770

[B22] Fetter K. Van Wilder V. Moshelion M. Chaumont F. (2004). Interactions between plasma membrane aquaporins modulate their water channel activity. *Plant Cell* 16 215–228. 10.1105/tpc.017194 14671024 PMC301406

[B23] Ghorbani A. Omran V. Razavi S. M. Pirdashti H. Ranjbar M. (2019). Piriformospora indica confers salinity tolerance on tomato (*Lycopersicon esculentum* Mill.) through amelioration of nutrient accumulation, K+/Na+ homeostasis and water status. *Plant Cell Rep.* 38 1151–1163. 10.1007/s00299-019-02434-w 31152194

[B24] Gomes D. Agasse A. Thiébaud P. Delrot S. Gerós H. Chaumont F. (2009). Aquaporins are multifunctional water and solute transporters highly divergent in living organisms. *Biochim. Biophys. Acta* 1788 1213–1228. 10.1016/j.bbamem.2009.03.009 19327343

[B25] Guo L. Wang Z. Lin H. Cui W. Chen J. Liu M. (2006). Expression and functional analysis of the rice plasma-membrane intrinsic protein gene family. *Cell Res.* 16 277–286. 10.1038/sj.cr.7310035 16541126

[B26] Hachez C. Besserer A. Chevalier A. S. Chaumont F. (2013). Insights into plant plasma membrane aquaporin trafficking. *Trends Plant Sci.* 18 344–352. 10.1016/j.tplants.2012.12.003 23291163

[B27] Horie T. Kaneko T. Sugimoto G. Sasano S. Panda S. K. Shibasaka M. (2011). Mechanisms of water transport mediated by PIP aquaporins and their regulation via phosphorylation events under salinity stress in barley roots. *Plant Cell Physiol.* 52 663–675. 10.1093/pcp/pcr027 21441236

[B28] Hu S. Zhou Q. An J. Yu B. (2016). Cloning PIP genes in drought tolerant vetiver grass and responses of transgenic VzPIP2;1 soybean plants to water stress. *Biol. Plant.* 60 655–666. 10.1007/s10535-016-0631-5

[B29] Hu W. Yuan Q. Wang Y. Cai R. Deng X. Wang J. (2012). Overexpression of a wheat aquaporin gene, TaAQP8, enhances salt stress tolerance in transgenic tobacco. *Plant Cell Physiol.* 53 2127–2141. 10.1093/pcp/pcs154 23161856

[B30] Huang X. Cao L. Fan J. Ma G. Chen L. (2022). CdWRKY2-mediated sucrose biosynthesis and CBF-signalling pathways coordinately contribute to cold tolerance in bermudagrass. *Plant Biotechnol. J.* 20 660–675. 10.1111/pbi.13745 34743386 PMC8989505

[B31] Hussain A. Tanveer R. Mustafa G. Farooq M. Amin I. Mansoor S. (2020). Comparative phylogenetic analysis of aquaporins provides insight into the gene family expansion and evolution in plants and their role in drought tolerant and susceptible chickpea cultivars. *Genomics* 112 263–275. 10.1016/j.ygeno.2019.02.005 30826442

[B32] Jian B. Hou W. Wu C. Liu B. Liu W. Song S. (2009). Agrobacterium rhizogenes-mediated transformation of superroot-derived *Lotus corniculatus* plants: a valuable tool for functional genomics. *BMC Plant Biol.* 9:78. 10.1186/1471-2229-9-78 19555486 PMC2708162

[B33] Jiang C. Song X. He H. Chu L. Zhou H. Zhao Y. (2020). Genome-wide identification of plasma membrane aquaporin gene family in populus and functional identification of PIP1;1 involved in osmotic stress. *Environ. Exp. Bot.* 179:104200. 10.1016/j.envexpbot.2020.104200

[B34] Johanson U. Karlsson M. Johansson I. Gustavsson S. Sjövall S. Fraysse L. (2001). The complete set of genes encoding major intrinsic proteins in *Arabidopsis* provides a framework for a new nomenclature for major intrinsic proteins in plants. *Plant Physiol.* 126 1358–1369. 10.1104/pp.126.4.1358 11500536 PMC117137

[B35] Kapilan R. Vaziri M. Zwiazek J. J. (2018). Regulation of aquaporins in plants under stress. *Biol. Res.* 51:4. 10.1186/s40659-018-0152-0 29338771 PMC5769316

[B36] Kayum M. A. Park J. I. Nath U. K. Biswas M. K. Kim H. T. Nou I. S. (2017). Genome-wide expression profiling of aquaporin genes confer responses to abiotic and biotic stresses in *Brassica rapa*. *BMC Plant Biol.* 17:23. 10.1186/s12870-017-0979-5 28122509 PMC5264328

[B37] Laur J. Hacke U. G. (2014). Exploring *Picea glauca* aquaporins in the context of needle water uptake and xylem refilling. *New Phytol.* 203 388–400. 10.1111/nph.12806 24702644

[B38] Lescot M. Déhais P. Thijs G. Marchal K. Moreau Y. Van de Peer Y. (2002). PlantCARE, a database of plant cis-acting regulatory elements and a portal to tools for in silico analysis of promoter sequences. *Nucleic Acids Res.* 30 325–327. 10.1093/nar/30.1.325 11752327 PMC99092

[B39] Lin R. Zheng J. Pu L. Wang Z. Mei Q. Zhang M. (2021). Genome-wide identification and expression analysis of aquaporin family in *Canavalia rosea* and their roles in the adaptation to saline-alkaline soils and drought stress. *BMC Plant Biol.* 21:333. 10.1186/s12870-021-03034-1 34256694 PMC8278772

[B40] Liu H. Yang L. Xin M. Ma F. Liu J. (2019). Gene-wide analysis of aquaporin gene family in *Malus domestica* and heterologous expression of the gene MpPIP2;1 confers drought and salinity tolerance in *Arabidposis thaliana*. *Int. J. Mol. Sci.* 20:3710. 10.3390/ijms20153710 31362376 PMC6696234

[B41] Livak K. J. Schmittgen T. D. (2001). Analysis of relative gene expression data using real-time quantitative PCR and the 2(-Delta Delta C(T)) Method. *Methods* 25 402–408. 10.1006/meth.2001.1262 11846609

[B42] Ma P. Liu J. (2012). Isolation and characterization of a novel plasma membrane intrinsic protein gene, LcPIP1, in *Leymus chinensis* that enhances salt stress tolerance in *Saccharomyces cerevisiae*. *Appl. Biochem. Biotechnol.* 166 479–485. 10.1007/s12010-011-9443-4 22072142

[B43] Martinez-Ballesta M. C. Carvajal M. (2014). New challenges in plant aquaporin biotechnology. *Plant Sci.* 217-218 71–77. 10.1016/j.plantsci.2013.12.006 24467898

[B44] Mitsui Y. Shimomura M. Komatsu K. Namiki N. Shibata-Hatta M. Imai M. (2015). The radish genome and comprehensive gene expression profile of tuberous root formation and development. *Sci. Rep.* 5:10835. 10.1038/srep10835 26056784 PMC4650646

[B45] Moshelion M. Halperin O. Wallach R. Oren R. Way D. A. (2015). Role of aquaporins in determining transpiration and photosynthesis in water-stressed plants: crop water-use efficiency, growth and yield. *Plant Cell Environ.* 38 1785–1793. 10.1111/pce.12410 25039365

[B46] Murata K. Mitsuoka K. Hirai T. Walz T. Agre P. Heymann J. (2000). Structural determinants of water permeation through aquaporin-1. *Nature* 407 599–605. 10.1038/35036519 11034202

[B47] Muries B. Faize M. Carvajal M. Martínez-Ballesta M. C. (2011). Identification and differential inductio4n of the expression of aquaporins by salinity in broccoli plants. *Mol. Biosyst.* 7 1322–1335. 10.1039/c0mb00285b 21321750

[B48] Muto N. Komatsu K. Matsumoto T. (2021). Efficient Agrobacterium-mediated genetic transformation method using hypocotyl explants of radish (*Raphanus sativus* L.). *Plant Biotechnol.* 38 457–461. 10.5511/plantbiotechnology.21.1021b 35087312 PMC8761586

[B49] Nam M. H. Bang E. Kwon T. Y. Kim Y. Kim E. Cho K. (2015). Metabolite profiling of diverse rice germplasm and identification of conserved metabolic markers of rice roots in response to long-term mild salinity stress. *Int. J. Mol. Sci.* 16 21959–21974. 10.3390/ijms160921959 26378525 PMC4613291

[B50] Nguyen M. X. Moon S. Jung K. H. (2013). Genome-wide expression analysis of rice aquaporin genes and development of a functional gene network mediated by aquaporin expression in roots. *Planta* 238 669–681. 10.1007/s00425-013-1918-9 23801298

[B51] Ozu M. Galizia L. Acuña C. Amodeo G. (2018). Aquaporins: more than functional monomers in a tetrameric arrangement. *Cells* 7:209. 10.3390/cells7110209 30423856 PMC6262540

[B52] Pawłowicz I. Masajada K. (2019). Aquaporins as a link between water relations and photosynthetic pathway in abiotic stress tolerance in plants. *Gene* 687 166–172. 10.1016/j.gene.2018.11.031 30445023

[B53] Pawłowicz I. Rapacz M. Perlikowski D. Gondek K. Kosmala A. (2017). Abiotic stresses influence the transcript abundance of PIP and TIP aquaporins in *Festuca species*. *J. Appl. Genet.* 58 421–435. 10.1007/s13353-017-0403-8 28779288 PMC5655603

[B54] Qin Y. Wang D. Fu J. Zhang Z. Qin Y. Hu G. (2021). Agrobacterium rhizogenes-mediated hairy root transformation as an efficient system for gene function analysis in *Litchi chinensis*. *Plant Methods* 17:103. 10.1186/s13007-021-00802-w 34627322 PMC8502350

[B55] Ren X. Y. Vorst O. Fiers M. W. Stiekema W. J. Nap J. P. (2006). In plants, highly expressed genes are the least compact. *Trends Genet.* 22 528–532. 10.1016/j.tig.2006.08.008 16934358

[B56] Reuscher S. Akiyama M. Mori C. Aoki K. Shibata D. Shiratake K. (2013). Genome-wide identification and expression analysis of aquaporins in tomato. *PLoS One* 8:e79052. 10.1371/journal.pone.0079052 24260152 PMC3834038

[B57] Rose A. B. (2008). Intron-mediated regulation of gene expression. *Curr. Top. Microbiol. Immunol.* 326 277–290. 10.1007/978-3-540-76776-3_15 18630758

[B58] Shivaraj S. M. Deshmukh R. Bhat J. A. Sonah H. Bélanger R. R. (2017). Understanding aquaporin transport system in eelgrass (*Zostera marina* L.), an aquatic plant species. *Front. Plant Sci.* 8:1334. 10.3389/fpls.2017.01334 28824671 PMC5541012

[B59] Srivastava A. K. Penna S. Nguyen D. V. Tran L. S. (2016). Multifaceted roles of aquaporins as molecular conduits in plant responses to abiotic stresses. *Crit. Rev. Biotechnol.* 36 389–398. 10.3109/07388551.2014.973367 25430890

[B60] Sun X. Xu L. Wang Y. Luo X. Zhu X. Kinuthia K. B. (2016). Transcriptome-based gene expression profiling identifies differentially expressed genes critical for salt stress response in radish (*Raphanus sativus* L.). *Plant Cell Rep.* 35 329–346. 10.1007/s00299-015-1887-5 26518430

[B61] Tanaka N. Hayakawa M. Mano Y. Ohkawa H. Matsui C. (1985). Infection of turnip and radish storage roots with *Agrobacterium rhizogenes*. *Plant Cell Rep.* 4 74–77. 10.1007/BF00269210 24253688

[B62] Tang H. Yu Q. Li Z. Liu F. Su W. Zhang C. (2021). A PIP-mediated osmotic stress signaling cascade plays a positive role in the salt tolerance of sugarcane. *BMC Plant Biol.* 21:589. 10.1186/s12870-021-03369-9 34903178 PMC8667355

[B63] Tuteja N. (2007). Mechanisms of high salinity tolerance in plants. *Methods Enzymol.* 428 419–438. 10.1016/S0076-6879(07)28024-3 17875432

[B64] Tyerman S. D. Bohnert H. J. Maurel C. Steudle E. Smith J. A. C. (1999). Plant aquaporins: their molecular biology, biophysics and significance for plant water relations. *J. Exp. Bot.* 50 1055–1071. 10.1054/ceca.2001.0254 11990298

[B65] Ueda M. Tsutsumi N. Fujimoto M. (2016). Salt stress induces internalization of plasma membrane aquaporin into the vacuole in *Arabidopsis thaliana*. *Biochem. Biophys. Res. Commun.* 474 742–746. 10.1016/j.bbrc.2016.05.028 27163638

[B66] Wei P. Wang L. Liu A. Yu B. Lam H. M. (2016). GmCLC1 confers enhanced salt tolerance through regulating chloride accumulation in soybean. *Front. Plant Sci.* 7:1082. 10.3389/fpls.2016.01082 27504114 PMC4959425

[B67] Xie Y. Ye S. Wang Y. Xu L. Zhu X. Yang J. (2015). Transcriptome-based gene profiling provides novel insights into the characteristics of radish root response to Cr stress with next-generation sequencing. *Front. Plant Sci.* 6:202. 10.3389/fpls.2015.00202 25873924 PMC4379753

[B68] Xu B. Sathitsuksanoh N. Tang Y. Udvardi M. K. Zhang J. Shen Z. (2012). Overexpression of AtLOV1 in Switchgrass alters plant architecture, lignin content, and flowering time. *PLoS One* 7:e47399. 10.1371/journal.pone.0047399 23300513 PMC3530547

[B69] Xu L. Wang Y. Zhai L. Xu Y. Wang L. Zhu X. (2013). Genome-wide identification and characterization of cadmium-responsive microRNAs and their target genes in radish (*Raphanus sativus* L.) roots. *J. Exp. Bot.* 64 4271–4287. 10.1093/jxb/ert240 24014874 PMC3808317

[B70] Yu Y. Xuan Y. Bian X. Zhang L. Pan Z. Kou M. (2020). Overexpression of phosphatidylserine synthase IbPSS1 affords cellular Na+ homeostasis and salt tolerance by activating plasma membrane Na+/H+ antiport activity in sweet potato roots. *Hortic. Res.* 7:131. 10.1038/s41438-020-00358-1 32821414 PMC7395154

[B71] Zargar S. M. Nagar P. Deshmukh R. Nazir M. Wani A. A. Masoodi K. Z. (2017). Aquaporins as potential drought tolerance inducing proteins: towards instigating stress tolerance. *J. Proteomics* 169 233–238. 10.1016/j.jprot.2017.04.010 28412527

[B72] Zhang W. Li J. Dong J. Wang Y. Xu L. Li K. (2021). RsSOS1 responding to salt stress might be involved in regulating salt tolerance by maintaining Na^+^ homeostasis in radish (*Raphanus sativus* L.) *Horticulturae*, 7:458. 10.3390/horticulturae7110458

[B73] Zhang Y. Fang Q. Zheng J. Li Z. Li Y. Feng Y. (2022). GmLecRlk, a lectin receptor-like protein kinase, contributes to salt stress tolerance by regulating salt-responsive genes in soybean. *Int. J. Mol. Sci.* 23:1030. 10.3390/ijms23031030 35162952 PMC8835537

[B74] Zhou H. Xiao F. Zheng Y. Liu G. Zhuang Y. Wang Z. (2022). PAMP-INDUCED SECRETED PEPTIDE 3 modulates salt tolerance through RECEPTOR-LIKE KINASE 7 in plants. *Plant Cell* 34 927–944. 10.1093/plcell/koab292 34865139 PMC8824610

[B75] Zhou L. Wang C. Liu R. Han Q. Vandeleur R. K. Du J. (2014). Constitutive overexpression of soybean plasma membrane intrinsic protein GmPIP1;6 confers salt tolerance. *BMC Plant Biol.* 14:181. 10.1186/1471-2229-14-181 24998596 PMC4105146

[B76] Zhu J. (2016). Abiotic stress signaling and responses in plants. *Cell* 167 313–324. 10.1016/j.cell.2016.08.029 27716505 PMC5104190

