# Supplementary material for: Genome-Wide Characterization of the Aquaporin Gene Family in Radish and Functional Analysis of RsPIP2-6 Involved in Salt Stress
Source: Front Plant Sci. 2022 Jul 13;13:860742. doi: 10.3389/fpls.2022.860742 (PMC9337223; doi:10.3389/fpls.2022.860742)
Supplement: Supplementary file 1 [file Table_1.DOCX]

| **Table S1. Number of transmembrane regions in RsAQP proteins** | |
| --- | --- |
| Protein name | Transmembrane region number |
| RsPIP1-1 | 6 |
| RsPIP1-2 | 6 |
| RsPIP1-3 | 6 |
| RsPIP1-4 | 6 |
| RsPIP1-5 | 6 |
| RsPIP1-6 | 6 |
| RsPIP1-7 | 6 |
| RsPIP2-1 | 6 |
| RsPIP2-2 | 6 |
| RsPIP2-3 | 6 |
| RsPIP2-4 | 6 |
| RsPIP2-5 | 6 |
| RsPIP2-6 | 6 |
| RsPIP2-7 | 6 |
| RsPIP2-8 | 6 |
| RsPIP2-9 | 6 |
| RsPIP2-10 | 6 |
| RsPIP2-11 | 4 |
| RsPIP2-12 | 6 |
| RsPIP2-13 | 6 |
| RsPIP2-14 | 6 |
| RsNIP1-1 | 5 |
| RsNIP1-2 | 5 |
| RsNIP1-3 | 5 |
| RsNIP2-1 | 4 |
| RsNIP2-2 | 5 |
| RsNIP2-3 | 5 |
| RsNIP4-1 | 6 |
| RsNIP4-2 | 6 |
| RsNIP4-3 | 6 |
| RsNIP4-4 | 6 |
| RsNIP5-1 | 5 |
| RsNIP6-1 | 6 |
| RsNIP6-2 | 5 |
| RsNIP6-3 | 5 |
| RsNIP7-1 | 3 |
| RsNIP7-2 | 3 |
| RsTIP1-1 | 7 |
| RsTIP1-2 | 7 |
| RsTIP1-3 | 7 |
| RsTIP1-4 | 7 |
| RsTIP1-5 | 6 |
| RsTIP1-6 | 7 |
| RsTIP2-1 | 6 |
| RsTIP2-2 | 6 |
| RsTIP2-3 | 6 |
| RsTIP2-4 | 7 |
| RsTIP2-5 | 5 |
| RsTIP2-6 | 3 |
| RsTIP2-7 | 4 |
| RsTIP2-8 | 12 |
| RsTIP3-1 | 6 |
| RsTIP3-2 | 6 |
| RsTIP3-3 | 5 |
| RsTIP4-1 | 7 |
| RsTIP5-1 | 6 |
| RsSIP1-1 | 5 |
| RsSIP1-2 | 5 |
| RsSIP2-1 | 6 |
| RsSIP2-2 | 5 |
| RsSIP2-3 | 6 |
