# Supplementary material for: Genome-Wide Characterization of the Aquaporin Gene Family in Radish and Functional Analysis of RsPIP2-6 Involved in Salt Stress
Source: Front Plant Sci. 2022 Jul 13;13:860742. doi: 10.3389/fpls.2022.860742 (PMC9337223; doi:10.3389/fpls.2022.860742)
Supplement: Supplementary file 2 [file Table_2.DOCX]

| **Table S2. Chromosomal localization of *RsAQP* genes in radish** | | |
| --- | --- | --- |
| Gene name | Chromosome | Location（bp） |
| *RsPIP1-1* | R5 | 31,564,129-31,565,538 |
| *RsPIP1-2* | R4 | 49,463,311-49,464,680 |
| *RsPIP1-3* | R4 | 42,737,923-42,739,152 |
| *RsPIP1-4* | R4 | 42,660,698-42,661,927 |
| *RsPIP1-5* | R1 | 328,718-329,870 |
| *RsPIP1-6* | R9 | 23,937,940-23,939,187 |
| *RsPIP1-7* | R4 | 5,657,722-5,658,840 |
| *RsPIP2-1* | R6 | 48,899,567-48,900,700 |
| *RsPIP2-2* | R6 | 48,918,497-48,919,630 |
| *RsPIP2-3* | R6 | 48,904,594-48,905,751 |
| *RsPIP2-4* | R6 | 48,923,524-48,924,681 |
| *RsPIP2-5* | R3 | 16,802,109-16,803,257 |
| *RsPIP2-6* | R5 | 26,802,439-26,803,943 |
| *RsPIP2-7* | R7 | 1,395,675-1,397,313 |
| *RsPIP2-8* | R2 | 22,310,787-22,312,232 |
| *RsPIP2-9* | R3 | 23,356,442-23,357,864 |
| *RsPIP2-10* | R3 | 15,631,903-15,634,597 |
| *RsPIP2-11* | R5 | 28,285,890-28,286,874 |
| *RsPIP2-12* | R4 | 1,521,176-1,522,477 |
| *RsPIP2-13* | R2 | 41,819,425-41,820,720 |
| *RsPIP2-14* | R8 | 13,082,546-13,084,097 |
| *RsTIP1-1* | R4 | 38,616,846-38,617,779 |
| *RsTIP1-2* | R4 | 15,628,920-15,629,991 |
| *RsTIP1-3* | R6 | 17,696,816-17,698,020 |
| *RsTIP1-4* | R6 | 17,668,304-17,669,508 |
| *RsTIP1-5* | R3 | 632,067-632,825 |
| *RsTIP1-6* | R9 | 23,317,338-23,318,096 |
| *RsTIP2-1* | R5 | 7,587,285-7,588,676 |
| *RsTIP2-2* | R6 | 6,180,568-6,181,897 |
| *RsTIP2-3* | R6 | 6,196,828-6,198,157 |
| *RsTIP2-4* | R5 | 41,925,224-41,926,637 |
| *RsTIP2-5* | R1 | 24,550,361-24,551,487 |
| *RsTIP2-6* | R4 | 18,118,616-18,119,032 |
| *RsTIP2-7* | R6 | 20,139,630-20,141,502 |
| *RsTIP2-8* | R2 | 32,968,650-32,975,989 |
| *RsTIP3-1* | R9 | 5,424,917-5,426,104 |
| *RsTIP3-2* | R6 | 34,116,937-34,118,136 |
| *RsTIP3-3* | R1 | 7,300,617-7,301,771 |
| *RsTIP4-1* | R4 | 33,269,378-33,270,667 |
| *RsTIP5-1* | R6 | 39,078,903-39,079,859 |
| *RsNIP1-1* | R2 | 33,934,804-33,943,803 |
| *RsNIP1-2* | R2 | 33,959,479-33,968,478 |
| *RsNIP1-3* | R4 | 7,215,242-7,217,282 |
| *RsNIP2-1* | R5 | 25,631,351-25,633,534 |
| *RsNIP2-2* | R8 | 19,840,910-19,842,819 |
| *RsNIP2-3* | R5 | 21,017,207-21,019,044 |
| *RsNIP4-1* | R4 | 25,830,157-25,831,627 |
| *RsNIP4-2* | RUS | 41,948-43,294 |
| *RsNIP4-3* | RUS | 4,083-5,296 |
| *RsNIP4-4* | RUS | 839-2,099 |
| *RsNIP5-1* | R2 | 11,159,921-11,163,442 |
| *RsNIP6-1* | R2 | 371,582-373,086 |
| *RsNIP6-2* | R2 | 418,245-419,333 |
| *RsNIP6-3* | R2 | 398,569-406,203 |
| *RsNIP7-1* | R5 | 2,293,981-2,294,613 |
| *RsNIP7-2* | R5 | 2,298,831-2,299,513 |
| *RsSIP1-1* | R5 | 1,230,060-1,231,582 |
| *RsSIP1-2* | R6 | 1,032,748-1,034,336 |
| *RsSIP2-1* | R5 | 29,559,646-29,560,568 |
| *RsSIP2-2* | R7 | 20,324,926-20,326,302 |
| *RsSIP2-3* | RUS | 143,802-145,407 |
