# Supplementary material for: Genome-Wide Characterization of the Aquaporin Gene Family in Radish and Functional Analysis of RsPIP2-6 Involved in Salt Stress
Source: Front Plant Sci. 2022 Jul 13;13:860742. doi: 10.3389/fpls.2022.860742 (PMC9337223; doi:10.3389/fpls.2022.860742)
Supplement: Supplementary file 3 [file Table_3.DOCX]

**Table S3 Primer sequences used for plasmid construction and RT-qPCR analysis**

| Plasmid Construction Primers | Sequence |
| --- | --- |
| *RsPIP2-6*-F-*Xba* I | atacaccaaatcgactctagaATGGCGAAGGACGTGGAAG |
| *RsPIP2-6*-R-*Kpn* I | gcccttgctcaccatggtaccGACGTTTGCAGCACTTCTGAAA |

| RT-qPCR Primers | Sequence |
| --- | --- |
| *RsActin*-F | 5'GCATCACACTTTCTACAAC3' |
| *RsActin*-R | 5'CCTGGATAGCAACATACAT3' |
| *RsPIP1-3*-F | 5'ATCGCCACGTTTCTGTTCCT3' |
| *RsPIP1-3*-R | 5'CAAACGTGACAGCTGGGTTG3' |
| *RsPIP1-6*-F | 5'ATCAACCCAGCGGTTACGTT3' |
| *RsPIP1-6*-R | 5'GCTGGAAACCCTTGACGACT3' |
| *RsPIP2-1*-F | 5'TCGGAACATTCGTCCTGGTC3' |
| *RsPIP2-1*-R | 5'AGATAACCGCGGCTCCAAAA3' |
| *RsPIP2-6*-F | 5'ACCATTCCCATCACCGGAAC3' |
| *RsPIP2-6*-R | 5'AACCTGAAGCCCTCAGAACG3' |
| *RsPIP2-10*-F | 5'CTTGCGGAGTTGGTTTGGTG3' |
| *RsPIP2-10*-R | 5'GAATGTGAGAGTCACGGGCA3' |
| *RsPIP2-13*-F | 5'GTCTTCTCTGCTACCGACCC3' |
| *RsPIP2-13*-R | 5'GGATGGTTGCCAAATGCACC3' |
| *RsPIP2-14*-F | 5'AGAGGGACACACTCAAAGCC3' |
| *RsPIP2-14*-R | 5'GCGATGGTGACGTAGAGGAA3' |
