# Supplementary material for: Genome-Wide Characterization of the Aquaporin Gene Family in Radish and Functional Analysis of RsPIP2-6 Involved in Salt Stress
Source: Front Plant Sci. 2022 Jul 13;13:860742. doi: 10.3389/fpls.2022.860742 (PMC9337223; doi:10.3389/fpls.2022.860742)
Supplement: Supplementary file 4 [file Table_4.DOCX]

| Gene (TRT12 in young root) | CK（0 h） | 6 h | 12 h | 24 h |
| --- | --- | --- | --- | --- |
| *RsPIP1-3* | 1.057883168 | 0.813190885 | 0.653467371 | 2.118110269 |
| *RsPIP1-6* | 1.042724967 | 0.370137817 | 0.299962447 | 0.572965807 |
| *RsPIP2-1* | 1.000843588 | 0.915854197 | 0.645592136 | 2.808075417 |
| *RsPIP2-6* | 1.060492855 | 0.780036667 | 1.118607551 | 5.856391203 |
| *RsPIP2-10* | 1.001152279 | 0.31183476 | 0.871743961 | 0.437790683 |
| *RsPIP2-13* | 1.020850789 | 0.704444301 | 0.450288339 | 0.714344037 |
| *RsPIP2-14* | 1.033748341 | 0.461138469 | 0.142683025 | 0.380154083 |
|  |  |  |  |  |
| Gene (TRT17 in young root) | CK（0 h） | 6 h | 12 h | 24 h |
| *RsPIP1-3* | 1.005093185 | 1.248712173 | 0.941558098 | 2.166164194 |
| *RsPIP1-6* | 1.00026124 | 2.995766256 | 2.46952161 | 2.236942986 |
| *RsPIP2-1* | 1.012763417 | 12.94890333 | 20.78951744 | 28.41921274 |
| *RsPIP2-6* | 1.024767503 | 12.40374898 | 15.5599539 | 28.84133338 |
| *RsPIP2-10* | 1.005366414 | 2.025612619 | 9.596312654 | 3.512856788 |
| *RsPIP2-13* | 1.008944203 | 2.619908797 | 1.299493007 | 1.02590608 |
| *RsPIP2-14* | 1.01592377 | 1.998266602 | 1.285671652 | 1.935815123 |
|  |  |  |  |  |
| Gene (TRT17 in taproot thickening period) | CK（0 h） | 6 h | 12 h | 24 h |
| *RsPIP1-3* | 1.005357634 | 3.260479322 | 0.608993505 | 2.297575066 |
| *RsPIP1-6* | 1.001983745 | 2.59746955 | 0.962268764 | 1.791648037 |
| *RsPIP2-1* | 1.007131988 | 8.78057854 | 4.64177315 | 13.46681175 |
| *RsPIP2-6* | 1.004601175 | 239.050533 | 1.894913463 | 135.7600677 |
| *RsPIP2-10* | 1.008936186 | 0.753885957 | 1.123309374 | 0.696633353 |
| *RsPIP2-13* | 1.040566637 | 0.763617446 | 0.939452523 | 1.084521281 |
| *RsPIP2-14* | 1.019274329 | 1.302693444 | 1.606855098 | 2.689104865 |

**Table S4. Expression of *RsPIP* genes at different times with 150 mM NaCl** **treatment**
