# Supplementary material for: Genome-Wide Characterization of the Aquaporin Gene Family in Radish and Functional Analysis of RsPIP2-6 Involved in Salt Stress
Source: Front Plant Sci. 2022 Jul 13;13:860742. doi: 10.3389/fpls.2022.860742 (PMC9337223; doi:10.3389/fpls.2022.860742)
Supplement: Supplementary file 5 [file Table_5.DOCX]

**Table S5 Relative expression raw data of young roots in TR12/TR17 and the taproot thickening period in TR17**

Raw data of young roots in TR12

| Pos | Sample Name | Type | Target Name | Cp |
| --- | --- | --- | --- | --- |
| A1 | CK | Reference Unknown | Actin | 15.62224895 |
| A2 | CK | Reference Unknown | Actin | 15.53028976 |
| A3 | CK | Reference Unknown | Actin | 15.24592235 |
| A4 | 6h | Reference Unknown | Actin | 14.51735941 |
| A5 | 6h | Reference Unknown | Actin | 13.96404671 |
| A6 | 6h | Reference Unknown | Actin | 14.66479199 |
| A7 | 12h | Reference Unknown | Actin | 14.5425855 |
| A8 | 12h | Reference Unknown | Actin | 14.40697397 |
| A9 | 12h | Reference Unknown | Actin | 13.66444528 |
| A10 | 24h | Reference Unknown | Actin | 14.38687121 |
| A11 | 24h | Reference Unknown | Actin | 14.59186565 |
| A12 | 24h | Reference Unknown | Actin | 13.99403528 |
| B1 | CK | Target Unknown | PIP1-3 | 15.42791946 |
| B2 | CK | Target Unknown | PIP1-3 | 15.33138003 |
| B3 | CK | Target Unknown | PIP1-3 | 14.38523622 |
| B4 | 6h | Target Unknown | PIP1-3 | 14.39576228 |
| B5 | 6h | Target Unknown | PIP1-3 | 14.45245216 |
| B6 | 6h | Target Unknown | PIP1-3 | 13.98540335 |
| B7 | 12h | Target Unknown | PIP1-3 | 14.45726465 |
| B8 | 12h | Target Unknown | PIP1-3 | 14.57450316 |
| B9 | 12h | Target Unknown | PIP1-3 | 14.19612142 |
| B10 | 24h | Target Unknown | PIP1-3 | 12.43824116 |
| B11 | 24h | Target Unknown | PIP1-3 | 12.84079926 |
| B12 | 24h | Target Unknown | PIP1-3 | 13.32613652 |
| C1 | CK | Target Unknown | PIP1-6 | 15.72885725 |
| C2 | CK | Target Unknown | PIP1-6 | 15.94836147 |
| C3 | CK | Target Unknown | PIP1-6 | 16.73485207 |
| C4 | 6h | Target Unknown | PIP1-6 | 16.38471515 |
| C5 | 6h | Target Unknown | PIP1-6 | 16.48191668 |
| C6 | 6h | Target Unknown | PIP1-6 | 16.60303873 |
| C7 | 12h | Target Unknown | PIP1-6 | 16.66798431 |
| C8 | 12h | Target Unknown | PIP1-6 | 16.34252307 |
| C9 | 12h | Target Unknown | PIP1-6 | 16.87981613 |
| C10 | 24h | Target Unknown | PIP1-6 | 15.66122073 |
| C11 | 24h | Target Unknown | PIP1-6 | 15.84442207 |
| C12 | 24h | Target Unknown | PIP1-6 | 15.9022881 |
| D1 | CK | Target Unknown | PIP2-1 | 17.57462791 |
| D2 | CK | Target Unknown | PIP2-1 | 17.71509883 |
| D3 | CK | Target Unknown | PIP2-1 | 17.67485518 |
| D4 | 6h | Target Unknown | PIP2-1 | 16.64454991 |
| D5 | 6h | Target Unknown | PIP2-1 | 16.63625482 |
| D6 | 6h | Target Unknown | PIP2-1 | 16.81924209 |
| D7 | 12h | Target Unknown | PIP2-1 | 17.39174903 |
| D8 | 12h | Target Unknown | PIP2-1 | 16.59298464 |
| D9 | 12h | Target Unknown | PIP2-1 | 17.21596447 |
| D10 | 24h | Target Unknown | PIP2-1 | 15.44066559 |
| D11 | 24h | Target Unknown | PIP2-1 | 14.84472914 |
| D12 | 24h | Target Unknown | PIP2-1 | 14.86073514 |
| E1 | CK | Target Unknown | PIP2-6 | 16.54310782 |
| E2 | CK | Target Unknown | PIP2-6 | 17.43046595 |
| E3 | CK | Target Unknown | PIP2-6 | 17.66383635 |
| E4 | 6h | Target Unknown | PIP2-6 | 16.25374715 |
| E5 | 6h | Target Unknown | PIP2-6 | 16.47894898 |
| E6 | 6h | Target Unknown | PIP2-6 | 16.77435515 |
| E7 | 12h | Target Unknown | PIP2-6 | 15.99612072 |
| E8 | 12h | Target Unknown | PIP2-6 | 15.90668986 |
| E9 | 12h | Target Unknown | PIP2-6 | 15.51221769 |
| E10 | 24h | Target Unknown | PIP2-6 | 13.46381101 |
| E11 | 24h | Target Unknown | PIP2-6 | 13.333353 |
| E12 | 24h | Target Unknown | PIP2-6 | 13.80454439 |
| F1 | CK | Target Unknown | PIP2-10 | 18.82006884 |
| F2 | CK | Target Unknown | PIP2-10 | 18.98754933 |
| F3 | CK | Target Unknown | PIP2-10 | 18.87801421 |
| F4 | 6h | Target Unknown | PIP2-10 | 19.3116022 |
| F5 | 6h | Target Unknown | PIP2-10 | 19.38686102 |
| F6 | 6h | Target Unknown | PIP2-10 | 19.83089736 |
| F7 | 12h | Target Unknown | PIP2-10 | 17.8954439 |
| F8 | 12h | Target Unknown | PIP2-10 | 18.21308837 |
| F9 | 12h | Target Unknown | PIP2-10 | 17.48072567 |
| F10 | 24h | Target Unknown | PIP2-10 | 19.18643354 |
| F11 | 24h | Target Unknown | PIP2-10 | 18.9940704 |
| F12 | 24h | Target Unknown | PIP2-10 | 18.69703125 |
| G1 | CK | Target Unknown | PIP2-13 | 17.82501157 |
| G2 | CK | Target Unknown | PIP2-13 | 18.33973364 |
| G3 | CK | Target Unknown | PIP2-13 | 17.6313403 |
| G4 | 6h | Target Unknown | PIP2-13 | 17.38022925 |
| G5 | 6h | Target Unknown | PIP2-13 | 17.31035061 |
| G6 | 6h | Target Unknown | PIP2-13 | 17.37056846 |
| G7 | 12h | Target Unknown | PIP2-13 | 17.73260391 |
| G8 | 12h | Target Unknown | PIP2-13 | 17.77587636 |
| G9 | 12h | Target Unknown | PIP2-13 | 17.96700811 |
| G10 | 24h | Target Unknown | PIP2-13 | 17.27100177 |
| G11 | 24h | Target Unknown | PIP2-13 | 17.20671165 |
| G12 | 24h | Target Unknown | PIP2-13 | 17.35229153 |
| H1 | CK | Target Unknown | PIP2-14 | 14.70715093 |
| H2 | CK | Target Unknown | PIP2-14 | 14.38750199 |
| H3 | CK | Target Unknown | PIP2-14 | 13.81701012 |
| H4 | 6h | Target Unknown | PIP2-14 | 14.05007359 |
| H5 | 6h | Target Unknown | PIP2-14 | 13.95839156 |
| H6 | 6h | Target Unknown | PIP2-14 | 15.3933234 |
| H7 | 12h | Target Unknown | PIP2-14 | 15.5944497 |
| H8 | 12h | Target Unknown | PIP2-14 | 15.77359042 |
| H9 | 12h | Target Unknown | PIP2-14 | 16.26760552 |
| H10 | 24h | Target Unknown | PIP2-14 | 14.59037899 |
| H11 | 24h | Target Unknown | PIP2-14 | 14.4612823 |
| H12 | 24h | Target Unknown | PIP2-14 | 14.6255784 |

Raw data of young roots in TR17

| Pos | Sample Name | Type | Target Name | Cp |
| --- | --- | --- | --- | --- |
| A1 | CK | Reference Unknown | actin | 15.42693094 |
| A2 | CK | Reference Unknown | actin | 15.4768604 |
| A3 | CK | Reference Unknown | actin | 14.80504536 |
| A4 | 6h | Reference Unknown | actin | 18.62418755 |
| A5 | 6h | Reference Unknown | actin | 17.80130452 |
| A6 | 6h | Reference Unknown | actin | 17.92036325 |
| A7 | 12h | Reference Unknown | actin | 17.81547399 |
| A8 | 12h | Reference Unknown | actin | 17.7826171 |
| A9 | 12h | Reference Unknown | actin | 17.89411658 |
| A10 | 24h | Reference Unknown | actin | 18.62517284 |
| A11 | 24h | Reference Unknown | actin | 17.7259791 |
| A12 | 24h | Reference Unknown | actin | 18.30045938 |
| B1 | CK | Target Unknown | PIP1-3 | 14.48683008 |
| B2 | CK | Target Unknown | PIP1-3 | 14.83426584 |
| B3 | CK | Target Unknown | PIP1-3 | 14.58066091 |
| B4 | 6h | Target Unknown | PIP1-3 | 17.47866201 |
| B5 | 6h | Target Unknown | PIP1-3 | 16.87165046 |
| B6 | 6h | Target Unknown | PIP1-3 | 17.29582323 |
| B7 | 12h | Target Unknown | PIP1-3 | 17.50036893 |
| B8 | 12h | Target Unknown | PIP1-3 | 17.6946775 |
| B9 | 12h | Target Unknown | PIP1-3 | 16.88050413 |
| B10 | 24h | Target Unknown | PIP1-3 | 16.51523098 |
| B11 | 24h | Target Unknown | PIP1-3 | 14.0093046 |
| B12 | 24h | Target Unknown | PIP1-3 | 16.48428212 |
| C1 | CK | Target Unknown | PIP1-6 | 17.3228584 |
| C2 | CK | Target Unknown | PIP1-6 | 17.39975626 |
| C3 | CK | Target Unknown | PIP1-6 | 17.3393988 |
| C4 | 6h | Target Unknown | PIP1-6 | 18.86491802 |
| C5 | 6h | Target Unknown | PIP1-6 | 18.34747003 |
| C6 | 6h | Target Unknown | PIP1-6 | 18.79413542 |
| C7 | 12h | Target Unknown | PIP1-6 | 18.63279395 |
| C8 | 12h | Target Unknown | PIP1-6 | 18.66917144 |
| C9 | 12h | Target Unknown | PIP1-6 | 18.6310445 |
| C10 | 24h | Target Unknown | PIP1-6 | 19.15785015 |
| C11 | 24h | Target Unknown | PIP1-6 | 19.18906417 |
| C12 | 24h | Target Unknown | PIP1-6 | 18.45667687 |
| D1 | CK | Target Unknown | PIP2-1 | 19.3120449 |
| D2 | CK | Target Unknown | PIP2-1 | 19.85883564 |
| D3 | CK | Target Unknown | PIP2-1 | 19.43911686 |
| D4 | 6h | Target Unknown | PIP2-1 | 18.58080119 |
| D5 | 6h | Target Unknown | PIP2-1 | 18.67522658 |
| D6 | 6h | Target Unknown | PIP2-1 | 18.9287519 |
| D7 | 12h | Target Unknown | PIP2-1 | 17.69174911 |
| D8 | 12h | Target Unknown | PIP2-1 | 17.81673203 |
| D9 | 12h | Target Unknown | PIP2-1 | 16.67606621 |
| D10 | 24h | Target Unknown | PIP2-1 | 17.55423786 |
| D11 | 24h | Target Unknown | PIP2-1 | 17.9867381 |
| D12 | 24h | Target Unknown | PIP2-1 | 17.56601514 |
| E1 | CK | Target Unknown | PIP2-6 | 17.42398299 |
| E2 | CK | Target Unknown | PIP2-6 | 16.97489181 |
| E3 | CK | Target Unknown | PIP2-6 | 17.75035361 |
| E4 | 6h | Target Unknown | PIP2-6 | 16.3026403 |
| E5 | 6h | Target Unknown | PIP2-6 | 16.76232326 |
| E6 | 6h | Target Unknown | PIP2-6 | 16.89150431 |
| E7 | 12h | Target Unknown | PIP2-6 | 16.53181682 |
| E8 | 12h | Target Unknown | PIP2-6 | 15.60261422 |
| E9 | 12h | Target Unknown | PIP2-6 | 14.71250346 |
| E10 | 24h | Target Unknown | PIP2-6 | 15.68637439 |
| E11 | 24h | Target Unknown | PIP2-6 | 15.51105727 |
| E12 | 24h | Target Unknown | PIP2-6 | 15.36251496 |
| F1 | CK | Target Unknown | PIP2-10 | 16.56888943 |
| F2 | CK | Target Unknown | PIP2-10 | 16.93475062 |
| F3 | CK | Target Unknown | PIP2-10 | 13.35824258 |
| F4 | 6h | Target Unknown | PIP2-10 | 18.55240075 |
| F5 | 6h | Target Unknown | PIP2-10 | 18.86529321 |
| F6 | 6h | Target Unknown | PIP2-10 | 18.45124529 |
| F7 | 12h | Target Unknown | PIP2-10 | 16.50250153 |
| F8 | 12h | Target Unknown | PIP2-10 | 16.23705362 |
| F9 | 12h | Target Unknown | PIP2-10 | 15.64797015 |
| F10 | 24h | Target Unknown | PIP2-10 | 18.5404119 |
| F11 | 24h | Target Unknown | PIP2-10 | 17.64376852 |
| F12 | 24h | Target Unknown | PIP2-10 | 17.73314553 |
| G1 | CK | Target Unknown | PIP2-13 | 15.32917612 |
| G2 | CK | Target Unknown | PIP2-13 | 15.44522298 |
| G3 | CK | Target Unknown | PIP2-13 | 14.9955766 |
| G4 | 6h | Target Unknown | PIP2-13 | 16.81833504 |
| G5 | 6h | Target Unknown | PIP2-13 | 16.51974843 |
| G6 | 6h | Target Unknown | PIP2-13 | 16.93238853 |
| G7 | 12h | Target Unknown | PIP2-13 | 17.2934742 |
| G8 | 12h | Target Unknown | PIP2-13 | 17.42403232 |
| G9 | 12h | Target Unknown | PIP2-13 | 17.73727434 |
| G10 | 24h | Target Unknown | PIP2-13 | 17.8212199 |
| G11 | 24h | Target Unknown | PIP2-13 | 18.51912686 |
| G12 | 24h | Target Unknown | PIP2-13 | 18.35717468 |
| H1 | CK | Target Unknown | PIP2-14 | 14.94991892 |
| H2 | CK | Target Unknown | PIP2-14 | 14.84109311 |
| H3 | CK | Target Unknown | PIP2-14 | 14.36819512 |
| H4 | 6h | Target Unknown | PIP2-14 | 16.68109059 |
| H5 | 6h | Target Unknown | PIP2-14 | 16.51758349 |
| H6 | 6h | Target Unknown | PIP2-14 | 16.60595316 |
| H7 | 12h | Target Unknown | PIP2-14 | 16.95048177 |
| H8 | 12h | Target Unknown | PIP2-14 | 17.13643805 |
| H9 | 12h | Target Unknown | PIP2-14 | 16.78896425 |
| H10 | 24h | Target Unknown | PIP2-14 | 16.50462524 |
| H11 | 24h | Target Unknown | PIP2-14 | 16.83178769 |
| H12 | 24h | Target Unknown | PIP2-14 | 16.94353678 |

Raw data of taproot thickening period in TR17

| Pos | Sample Name | Type | Target Name | Cp |
| --- | --- | --- | --- | --- |
| A1 | 24h | Reference Unknown | actin | 20.34172687 |
| A2 | 24h | Reference Unknown | actin | 20.80728092 |
| A3 | 24h | Reference Unknown | actin | 19.99003019 |
| A4 | 12h | Reference Unknown | actin | 20.49021296 |
| A5 | 12h | Reference Unknown | actin | 20.41767563 |
| A6 | 12h | Reference Unknown | actin | 20.67018832 |
| A7 | 6h | Reference Unknown | actin | 20.90957157 |
| A8 | 6h | Reference Unknown | actin | 20.90273816 |
| A9 | 6h | Reference Unknown | actin | 20.7202515 |
| A10 | 0h | Reference Unknown | actin | 20.51356976 |
| A11 | 0h | Reference Unknown | actin | 19.75239303 |
| A12 | 0h | Reference Unknown | actin | 20.20002322 |
| B1 | 24h | Target Unknown | PIP1-3 | 17.7044853 |
| B2 | 24h | Target Unknown | PIP1-3 | 17.77528889 |
| B3 | 24h | Target Unknown | PIP1-3 | 17.7067178 |
| B4 | 12h | Target Unknown | PIP1-3 | 19.5064153 |
| B5 | 12h | Target Unknown | PIP1-3 | 19.98276288 |
| B6 | 12h | Target Unknown | PIP1-3 | 19.93084638 |
| B7 | 6h | Target Unknown | PIP1-3 | 17.74083423 |
| B8 | 6h | Target Unknown | PIP1-3 | 17.87095329 |
| B9 | 6h | Target Unknown | PIP1-3 | 17.48001486 |
| B10 | 0h | Target Unknown | PIP1-3 | 18.57505295 |
| B11 | 0h | Target Unknown | PIP1-3 | 18.62156666 |
| B12 | 0h | Target Unknown | PIP1-3 | 18.91603958 |
| C1 | 24h | Target Unknown | PIP1-6 | 20.6639264 |
| C2 | 24h | Target Unknown | PIP1-6 | 22.06924957 |
| C3 | 24h | Target Unknown | PIP1-6 | 20.92822964 |
| C4 | 12h | Target Unknown | PIP1-6 | 22.41170258 |
| C5 | 12h | Target Unknown | PIP1-6 | 21.83664794 |
| C6 | 12h | Target Unknown | PIP1-6 | 22.25823203 |
| C7 | 6h | Target Unknown | PIP1-6 | 21.40018374 |
| C8 | 6h | Target Unknown | PIP1-6 | 20.38852435 |
| C9 | 6h | Target Unknown | PIP1-6 | 21.63112184 |
| C10 | 0h | Target Unknown | PIP1-6 | 21.84760388 |
| C11 | 0h | Target Unknown | PIP1-6 | 21.68394011 |
| C12 | 0h | Target Unknown | PIP1-6 | 21.63353036 |
| D1 | 24h | Target Unknown | PIP2-1 | 22.69605019 |
| D2 | 24h | Target Unknown | PIP2-1 | 23.40007763 |
| D3 | 24h | Target Unknown | PIP2-1 | 22.91724554 |
| D4 | 12h | Target Unknown | PIP2-1 | 25.33672624 |
| D5 | 12h | Target Unknown | PIP2-1 | 24.59260465 |
| D6 | 12h | Target Unknown | PIP2-1 | 24.24890539 |
| D7 | 6h | Target Unknown | PIP2-1 | 23.90014484 |
| D8 | 6h | Target Unknown | PIP2-1 | 23.86101533 |
| D9 | 6h | Target Unknown | PIP2-1 | 24.49223091 |
| D10 | 0h | Target Unknown | PIP2-1 | 26.63757333 |
| D11 | 0h | Target Unknown | PIP2-1 | 26.60698642 |
| D12 | 0h | Target Unknown | PIP2-1 | 26.26290625 |
| E1 | 24h | Target Unknown | PIP2-6 | 19.95194007 |
| E2 | 24h | Target Unknown | PIP2-6 | 19.5141267 |
| E3 | 24h | Target Unknown | PIP2-6 | 19.81910678 |
| E4 | 12h | Target Unknown | PIP2-6 | 26.68112878 |
| E5 | 12h | Target Unknown | PIP2-6 | 26.576937 |
| E6 | 12h | Target Unknown | PIP2-6 | 26.56581868 |
| E7 | 6h | Target Unknown | PIP2-6 | 20.35401437 |
| E8 | 6h | Target Unknown | PIP2-6 | 19.69192365 |
| E9 | 6h | Target Unknown | PIP2-6 | 19.87088696 |
| E10 | 0h | Target Unknown | PIP2-6 | 27.17717255 |
| E11 | 0h | Target Unknown | PIP2-6 | 27.31720281 |
| E12 | 0h | Target Unknown | PIP2-6 | 26.98103304 |
| F1 | 24h | Target Unknown | PIP2-10 | 22.22654237 |
| F2 | 24h | Target Unknown | PIP2-10 | 22.21430479 |
| F3 | 24h | Target Unknown | PIP2-10 | 22.32789462 |
| F4 | 12h | Target Unknown | PIP2-10 | 21.81685559 |
| F5 | 12h | Target Unknown | PIP2-10 | 21.71885109 |
| F6 | 12h | Target Unknown | PIP2-10 | 21.60904792 |
| F7 | 6h | Target Unknown | PIP2-10 | 22.31043285 |
| F8 | 6h | Target Unknown | PIP2-10 | 22.38692458 |
| F9 | 6h | Target Unknown | PIP2-10 | 23.32191919 |
| F10 | 0h | Target Unknown | PIP2-10 | 21.46447385 |
| F11 | 0h | Target Unknown | PIP2-10 | 21.29745074 |
| F12 | 0h | Target Unknown | PIP2-10 | 21.76650686 |
| G1 | 24h | Target Unknown | PIP2-13 | 18.81747211 |
| G2 | 24h | Target Unknown | PIP2-13 | 19.53356356 |
| G3 | 24h | Target Unknown | PIP2-13 | 19.51500335 |
| G4 | 12h | Target Unknown | PIP2-13 | 19.49881955 |
| G5 | 12h | Target Unknown | PIP2-13 | 19.60439001 |
| G6 | 12h | Target Unknown | PIP2-13 | 19.71023606 |
| G7 | 6h | Target Unknown | PIP2-13 | 19.88402725 |
| G8 | 6h | Target Unknown | PIP2-13 | 20.35291381 |
| G9 | 6h | Target Unknown | PIP2-13 | 20.49265319 |
| G10 | 0h | Target Unknown | PIP2-13 | 19.71452245 |
| G11 | 0h | Target Unknown | PIP2-13 | 18.98473522 |
| G12 | 0h | Target Unknown | PIP2-13 | 18.72403185 |
| H1 | 24h | Target Unknown | PIP2-14 | 19.29880228 |
| H2 | 24h | Target Unknown | PIP2-14 | 18.84593845 |
| H3 | 24h | Target Unknown | PIP2-14 | 18.88796483 |
| H4 | 12h | Target Unknown | PIP2-14 | 19.82790388 |
| H5 | 12h | Target Unknown | PIP2-14 | 19.8741182 |
| H6 | 12h | Target Unknown | PIP2-14 | 19.95956476 |
| H7 | 6h | Target Unknown | PIP2-14 | 20.40180482 |
| H8 | 6h | Target Unknown | PIP2-14 | 20.45356121 |
| H9 | 6h | Target Unknown | PIP2-14 | 20.68085183 |
| H10 | 0h | Target Unknown | PIP2-14 | 20.3174651 |
| H11 | 0h | Target Unknown | PIP2-14 | 19.81642501 |
| H12 | 0h | Target Unknown | PIP2-14 | 20.46524662 |
